# Automated segmentation of 3D cine cardiovascular magnetic resonance imaging

**DOI:** 10.3389/fcvm.2023.1167500

**Published:** 2023-10-13

**Authors:** Soroosh Tayebi Arasteh, Jennifer Romanowicz, Danielle F. Pace, Polina Golland, Andrew J. Powell, Andreas K. Maier, Daniel Truhn, Tom Brosch, Juergen Weese, Mahshad Lotfinia, Rob J. van der Geest, Mehdi H. Moghari

**Affiliations:** ^1^ Department of Cardiology, Boston Children’s Hospital, and Department of Pediatrics, Harvard Medical School, Boston, MA, United States; ^2^ Pattern Recognition Lab, Friedrich-Alexander-Universität Erlangen-Nürnberg, Erlangen, Germany; ^3^ Department of Diagnostic and Interventional Radiology, University Hospital RWTH Aachen, Aachen, Germany; ^4^ Department of Cardiology, Children’s Hospital Colorado, and School of Medicine, University of Colorado, Aurora, CO, United States; ^5^ Martinos Center for Biomedical Imaging, Massachusetts General Hospital, Charlestown, MA, United States; ^6^ Computer Science & Artificial Intelligence Lab, Massachusetts Institute of Technology, Cambridge, MA, United States; ^7^ Philips Research Laboratories, Hamburg, Germany; ^8^ Institute of Heat and Mass Transfer, RWTH Aachen University, Aachen, Germany; ^9^ Department of Radiology, Leiden University Medical Center, Leiden, Netherlands; ^10^ Department of Radiology, Children’s Hospital Colorado, and School of Medicine, University of Colorado, Aurora, CO, United States

**Keywords:** congenital heart disease, deep learning, 3D cine, CMR image analysis, automatic segmentation

## Abstract

**Introduction:**

As the life expectancy of children with congenital heart disease (CHD) is rapidly increasing and the adult population with CHD is growing, there is an unmet need to improve clinical workflow and efficiency of analysis. Cardiovascular magnetic resonance (CMR) is a noninvasive imaging modality for monitoring patients with CHD. CMR exam is based on multiple breath-hold 2-dimensional (2D) cine acquisitions that should be precisely prescribed and is expert and institution dependent. Moreover, 2D cine images have relatively thick slices, which does not allow for isotropic delineation of ventricular structures. Thus, development of an isotropic 3D cine acquisition and automatic segmentation method is worthwhile to make CMR workflow straightforward and efficient, as the present work aims to establish.

**Methods:**

Ninety-nine patients with many types of CHD were imaged using a non-angulated 3D cine CMR sequence covering the whole-heart and great vessels. Automatic supervised and semi-supervised deep-learning-based methods were developed for whole-heart segmentation of 3D cine images to separately delineate the cardiac structures, including both atria, both ventricles, aorta, pulmonary arteries, and superior and inferior vena cavae. The segmentation results derived from the two methods were compared with the manual segmentation in terms of Dice score, a degree of overlap agreement, and atrial and ventricular volume measurements.

**Results:**

The semi-supervised method resulted in a better overlap agreement with the manual segmentation than the supervised method for all 8 structures (Dice score 83.23 ± 16.76% vs. 77.98 ± 19.64%; *P*-value ≤0.001). The mean difference error in atrial and ventricular volumetric measurements between manual segmentation and semi-supervised method was lower (bias ≤ 5.2 ml) than the supervised method (bias ≤ 10.1 ml).

**Discussion:**

The proposed semi-supervised method is capable of cardiac segmentation and chamber volume quantification in a CHD population with wide anatomical variability. It accurately delineates the heart chambers and great vessels and can be used to accurately calculate ventricular and atrial volumes throughout the cardiac cycle. Such a segmentation method can reduce inter- and intra- observer variability and make CMR exams more standardized and efficient.

## 1. Introduction

Congenital heart disease (CHD) affects nearly *1%* of live births in the United States and Europe (1, 2). It is the leading cause of perinatal and infant death, and accounts for about *4%* of all neonatal deaths in the United States (3). Luckily, *83%* of babies with CHD now survive infancy and reach adulthood in the United States, due to medical, surgical, and intensive care advances (4). Despite these advances, there is no cure for CHD, and thus, these patients must be monitored lifelong. In addition to echocardiography, cardiovascular magnetic resonance (CMR) is one of the primary imaging modalities for monitoring CHD patients since it is non-invasive and does not require ionizing radiation (5). Although CMR can provide high spatial and temporal resolution images to calculate cardiac function, these measurements are traditionally based on multiple 2-dimensional (2D) cine image acquisitions that are precisely prescribed. For instance, 2D cine images are planned in a short-axis view to calculate cardiac function. However, defining the short-axis view is expert and institution dependent, and serial examinations may prescribe slightly different planes resulting in measurement variability. Furthermore, 2D cine images have a relatively large slice thickness which precludes isotropic delineation of the left and right ventricle for a more accurate measurement of cardiac function. An isotropic 3-dimensional (3D) cine sequence can mitigate these problems. Because it is non-angulated, prescription is independent from the expert and institution. Isotropic 3D cine images also allow for isotropic segmentation of the heart.

Manual segmentation of 3D cine images is, however, time consuming and impractical. Therefore, an automatic segmentation method is necessary to delineate the heart chambers and great vessels and to evaluate cardiac function. At present, artificial intelligence (AI) and, specifically, deep learning (DL) is making strong progress in automatic segmentation of CMR images (6–11). AI-based methods have been successfully utilized to delineate adult heart disease and CHD using 2D cine images (8, 10, 12). However, they are not designed for segmenting 3D cine CMR images. Prior work has also addressed the physical distortions and appearance changes that arise when segmenting the right and left ventricles in adult patients (7, 13–15). Method development regarding automated image analysis for CHD is very limited and has mostly been aimed towards computer-aided diagnosis (16–18), ventricular function quantification (19–23), 2D cine (9, 18, 24), or 3D static imaging (20, 25–29). Recently, more novel AI-based segmentation methods are proposed in the literature (30–35) which are based on vision transformers (36) but their performance on whole-heart segmentation have not been investigated. To date, there is no automatic DL-based segmentation method available that works on 3D cine datasets for delineating whole-heart and great vessels, nor is there work addressing cardiac segmentation in a wide range of CHD lesions.

In this article, we sought to develop fully automatic supervised and semi-supervised DL-based segmentation methods for a wide range of CHD lesions that delineate the heart and great vessels including left atrium (LA), left ventricle (LV), aorta (AO), superior vena cava (SVC), inferior vena cava (IVC), right atrium (RA), right ventricle (RV), and pulmonary arteries (PA), from 3D cine images. We hope the proposed technique enables isotropic delineation of cardiac anatomy and great vessels throughout the cardiac cycle and can be used for the assessment of cardiac function and anatomy.

## 2. Materials and methods

We have developed two 3D DL-based segmentation methods: a fully automatic supervised method and a fully automatic semi-supervised method. The objective was to evaluate and compare their performances in delineating the whole-heart and great vessels with respect to each other and to manual segmentation. Both methods employ the same network architecture and prediction pipeline; the only difference lies in their training processes.

### 2.1. The 3D cine datasets

#### 2.1.1. CMR imaging protocol and patient population

We retrospectively identified 99 patients who had undergone 3D cine CMR imaging at our institution. For each patient, clinically indicated 3D cine images were acquired about 2 min after injection of 0.15 mmol/kg of gadobutrol contrast agent using a 1.5 T Philips scanner (Philips, Best, The Netherlands). The imaging parameters for the 3D cine acquisition were as follows: field-of-view of 512 (frequency-encode) × 170 (phase-encode) × 170 (slice-encode) mm, Cartesian k-space trajectory, profile ordering centra, isotropic resolution of 1.51 × 1.48 × 1.51 mm reconstructed to 1.0 × 1.0 × 1.0 mm, 20 acquired heart phases reconstructed to 30 phases, parallel imaging technique (SENSE) with reduction factor of 2 (phase-encode) × 2 (slice-encode), TFE factor 11, and TFE shots 310, flip angle 60°, repetition time of 4.5 ms, echo time of 2.3 ms, nominal scan time of approximately 5:12 min assuming heartrate of 60 bpm and a 100% scan efficiency. A respiratory navigator was used for minimizing the respiratory motion of the heart. The images were reconstructed online on the scanner. After acquisition, the 3D cine images were de-identified for analysis. This retrospective study was reviewed and approved by the institutional review board of Boston Children's Hospital, Boston, MA, USA (IRB-P00011748).

The patient cohort had an age range of 0.8–72 years (median 16), heart rate range of 57–131 bpm (median 83), and weight range of 5.8–113.6 kg (median 55.3). The patient population was imaged to assess CHD (*n* = 81), acquired heart disease (*n* = 2), connective tissue disease or aortopathy (*n* = 6), cardiomyopathy (*n* = 5), arrhythmia (*n* = 3), or a family history of inheritable heart disease (*n* = 2). With consideration to what features of abnormal cardiac anatomy would make automatic segmentation of the heart more difficult, we devised a set of rules to categorize patients into 3 complexity classifications of normal, mild/moderate, and severe (Table 1).

**Table 1:** Method for classifying subjects’ cardiac anatomies into 3 categories of complexity. The 3 categories of complexity include normal anatomy and mild/moderate and severely complexity.

Category	Segmental anatomy	Ventricles	Shunts	Valve abnormalities	Miscellaneous
Normal	Normal	Two	Patent foramen ovale	Bicuspid aortic valve (BAV)	Cardiomyopathy, coarctation, interrupted aortic arch, aortopathy, anomalous coronaries
Mild/moderate	Double-outlet right ventricle (DORV), unrepaired D-TGA	Two/Two, but unequal	Arterial septal defect (ASD), ventricular septal defect (VSD), Patent Ductus Arteriosus (PDA), coronary fistula, aortopulmonary (AP) window, partial/total anomalous pulmonary venous connection (PAPVC/TAPVC)	Common atrioventricular valve (AVV), any valvular stenosis, any prosthetic valve, AVV straddle, Tetralogy of fallot (TOF), truncus, Ebstein	Dextro transposition of the great arteries (D-TGA) status post (S/P) arterial switch, S/P Glenn of Fontan, dextrocardia, 2× mild
Severe	Levo Transposition of the great arteries (L-TGA), more complex arrangements	One		Any valvular atresia	S/P atrial switch, 2× moderate

Table 2 illustrates further details about final patient categorization into normal anatomy, mild/moderate, and severely complex groups. Supplementary Table S2 provides detailed information about all the subjects in this study.

**Table 2:** Details of the subjects according to cardiac diagnosis.

Normal	26
Cardiomyopathy	5
Hypertrophic cardiomyopathy	3
Arrhythmogenic right ventricular cardiomyopathy (ARVC)	1
Left ventricular non-compaction cardiomyopathy	1
Arrhythmia	3
Rule out (R/O) ARVC	2
Wolff-Parkinson-White	1
Family history	2
Ventricular fibrillation arrest	1
Aortopathy	1
Coarctation/BAV	8
Isolated coarctation of the aorta (CoA)	3
CoA + BAV	2
Isolated BAV	3
Connective tissue disease (CTD)	6
Marfan's syndrome	4
CTD, aortopathy nitric oxide synthesis (NOS)	2
Miscellaneous	2
ARCAPA	1
Lipomatous hypertrophy of the interatrial septum	1
Mild/moderate	39
Left-to-right shunt	8
Repaired VSD + arch repair	3
Repaired ASD	1
Repaired TAPVC	1
Repaired AP window + arch repair	1
Left coronary artery to RV fistula	1
Sinus venosus defect	1
Prosthetic valve	4
Congenital aortic stenosis, S/P aortic valve replacement (AVR)	3
Rheumatic heart disease, S/P mitral valve regurgitation & AVR	1
Congenital aortic valve disease	9
Congenital pulmonary valve disease	2
D-TGA, S/P arterial switch operation	1
Repaired TOF	7
Repaired truncus arteriosus	2
Ebstein anomaly	2
DORV	2
D-TGA, S/P Rastelli repair	2
Severe	34
Single ventricle	5
Double-inlet left ventricle	2
Hypoplastic left heart syndrome, S/P Fontan	1
Tricuspid atresia S/P Fontan	1
Double-inlet/double-outlet RV	1
Complex segmental anatomy	3
Superior-inferior ventricles, S/P Glenn	2
Dextrocardia, D-TGA, crisscrossed AV valves, S/P Glenn	1
L-TGA	5
valvular atresia	11
TOF/PA	4
DORV/PA	4
PA/interventricular septum	2
Ebstein/PA	1
Atrial switch	2
2× Moderate Lesions	8
DORV, right-dominant atrioventricular canal	3
DORV, S/P Glenn	2
TOF, S/P Fontan	1
Dextrocardia, D-TGA	1
D-TGA, S/P Glenn	1

#### 2.1.2. Pre-processing

All the 3D frames of every 3D cine image were cropped around the heart so that all 30 frames were the same size for each patient. Per patient, a specific 3D bounding box containing the whole heart was visually chosen and the cropping occurred accordingly. This was done to lower computational costs and standardize the fields of view. Moreover, the frames corresponding to the end-diastole (ED) and end-systole (ES) were visually selected for each 3D cine dataset. Because intensity distributions vary across CMR images, intensity normalization is required. Therefore, we created an intensity normalization scheme based on the estimated blood pool and lung intensities in each image of the 3D cine dataset (24).

#### 2.1.3. Manual annotation and ground truth labels

From the 99 total cases analyzed in this study, 74 cases were utilized for training, 13 cases were chosen as validation images for optimizing the networks during the deep learning training, and 12 cases were selected as test images. The test and validation patients were selected in a balanced manner from the 3 anatomy complexity categories (normal, mild**/**moderate, and severe) to maintain generalizability as much as possible. Supplementary Table S1 shows the distribution of the 3D cine datasets based on their severity classes. The training and validation images were manually annotated on the ED and ES frames, i.e., only two out of thirty 3D frames contained manual annotations for training the DL-based segmentation methods. However, the test images were manually annotated on all 30 frames to serve as a reference, and to evaluate the DL-based segmentation methods throughout the cardiac cycle. A 3D U-Net (37, 38) convolutional neural network trained on 3D static CMR images (25, 25, 39) was used to pre-segment the ED and ES phases for the training and validation datasets. The output of the network was then refined using 3DSlicer toolkit (40) via generic segmentation tools such island relabeling, further painting, or erasing. The segmentation results were supervised and, if necessary, modified by a pediatric cardiologist with expertise in CMR imaging. A similar procedure was performed to segment all 30 phases of test datasets. Specifically, each of the 30 frames underwent pre-segmentation using the 3D U-Net convolutional neural network (37, 38) to segment all 30 phases of the test datasets individually. The pre-segmentation results were then manually adjusted for each phase by a single observer using 3DSlicer and its generic segmentation modules. Finally, a clinician reviewed and supervised the final segmentation results.

Two DL-based segmentation methods were developed for fully automatic segmentation of 3D cine images: 1. Supervised DL-based segmentation method and 2. Semi-supervised segmentation method. In the supervised DL-based segmentation method only the manual annotation of ED and ES frames were used for training. In the semi-supervised DL-based segmentation method, the manual annotation of ED and ES frames were automatically propagated to other 28 frames in the cardiac cycle, and manual annotation of ED and ES frames and propagated annotation of other 28 frames were used for training.

### 2.2. The supervised DL-based segmentation method

#### 2.2.1. Approach

**Figure 1:**
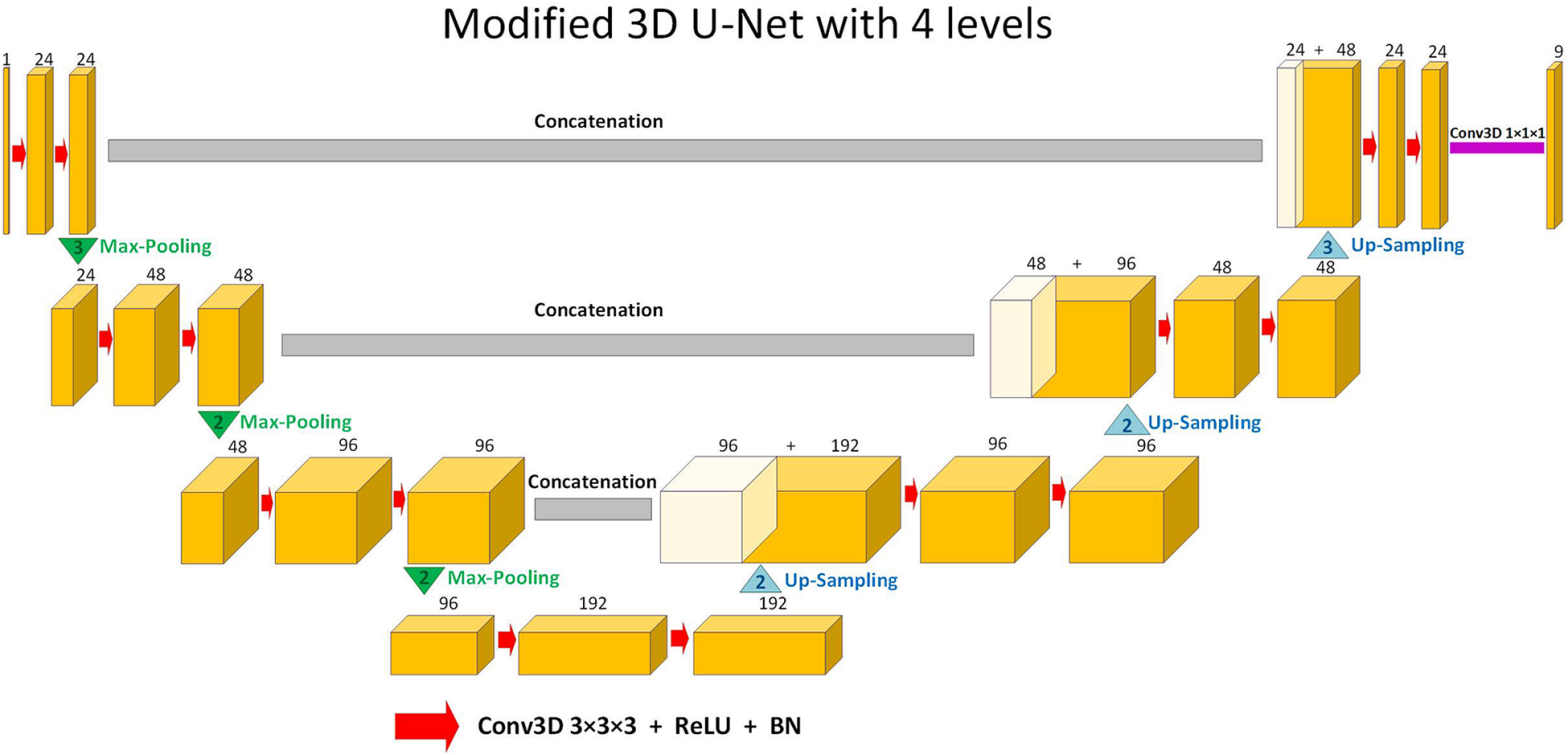
Schematic representation of the modified 3D U-Net (37,38) architecture used in this study for segmentation. Up-sampling is performed through nearest-neighbor interpolation. Each arrow denotes a 3 × 3 × 3 convolutional layer, subsequently followed by a rectified linear unit (ReLU) and batch normalization (BN). The channel numbers are mentioned on top of the feature maps. The concluding layer employs a 1 × 1 × 1 convolution to streamline to 9 output channels. These channels are then subjected to voxel-wise classification via a SoftMax layer.

The supervised DL-based segmentation method has a 3D U-Net structure (Figure 1). Like the standard U-Net structure (37) and benefiting from nnU-Net (41) configurations, it has a contracting and an expanding path, each with four resolution levels (37, 38). In the contracting path, each layer contains two 3 × 3 × 3 convolutions, each followed by a rectified linear unit (ReLU) (42), a batch normalization (43), and then a 2 × 2 × 2 max pooling layer with strides of two in each dimension. In the expanding path, each layer consists of a nearest neighbor up-sampling of 2 × 2 × 2 in each dimension, followed by two 3 × 3 × 3 convolutions each followed by a ReLU and a batch normalization layer. Shortcut connections from layers of equal resolution in the contracting path provide high-resolution features to the expanding path. In the last layer, a 1 × 1 × 1 convolution, which reduces the number of output channels to the number of labels, followed by a SoftMax layer, is used for the voxel-wise classification.

#### 2.2.2. Supervised training

We followed a supervised approach and solely utilized the manually annotated ED and ES frames of the 3D cine datasets for training. The model was optimized using the AMSGrad (44) optimizer with a learning rate of 3 × 10^−4^. As loss function, we chose the weighted cross-entropy with inverted class frequencies of the training data as loss weights to counteract the imbalanced class frequencies, with higher weights for smaller structures. To minimize the overhead and make maximum use of the graphics processing unit memory, we favored large input tiles over a large batch size and reduce the batch to a single 3D image (37). Consequently, the batch normalization acted like instance normalization in our implementation. We performed data augmentation by applying random affine transformations including rotation, shearing, translation, intensity scaling, and nonlinear transformations including left-right and anteroposterior flips (which are helpful for dextrocardia and other cardiac malposition in CHD), constant intensity shifts, and additive Gaussian noise on the datasets.

### 2.3. The semi-supervised DL-based segmentation method

#### 2.3.1. Approach

In each 3D cine dataset, only the ED and the ES phases of every 3D cine training image were manually annotated. Successive frames were not very different from each other. As such, inspired by Fan et al. (45), we utilized a label propagation algorithm to generate pseudo-labels for the intermediate frames which were not manually annotated to enhance our training. For each 3D cine image, we trained a separate DL-based segmentation neural network from scratch, using the ED and ES labels. We called this type of DL network the *propagator* network. This network was responsible for generating pseudo-labels for the remaining unlabeled 28 phases of the cine images.

**Figure 2:**
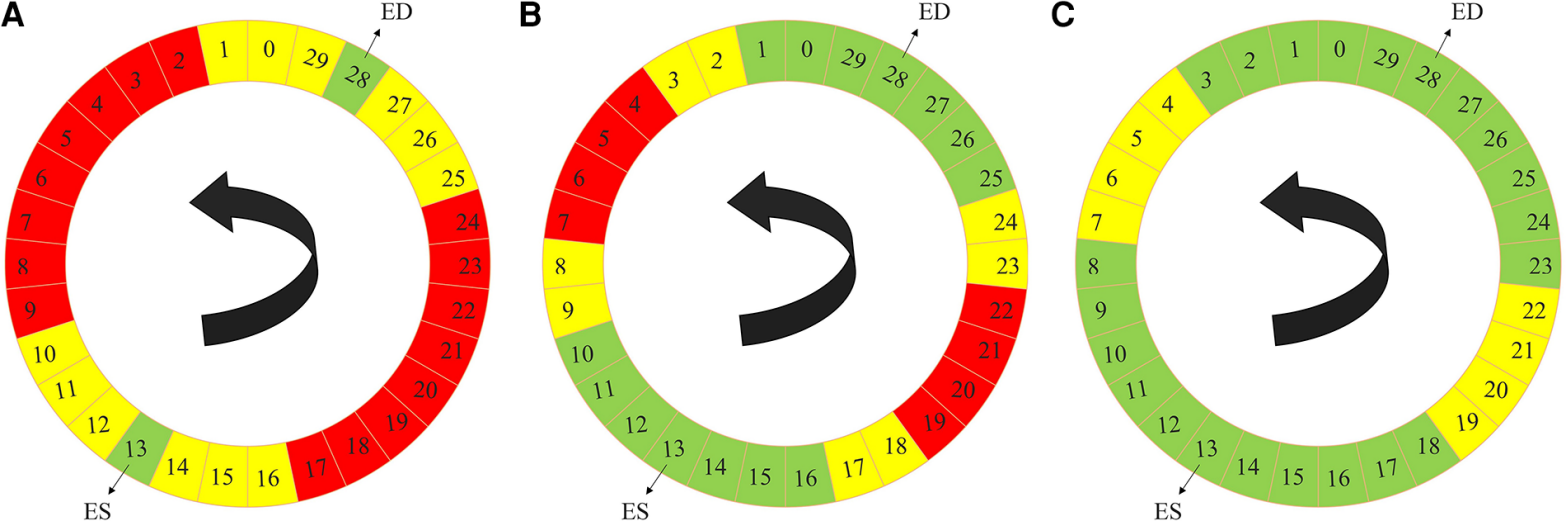
Label propagation process, to generate pseudo-labels for all the intermediate non-annotated frames to be used during weighted-probabilistic training, described by using one of the 3D cine datasets. (**A**) In the first step, the 3-nearest neighbors of the ED volume (29, 0, 1, 27, 26, and 25) and ES volume (14, 15, 16, 12, 11, and 10), shown in yellow, are segmented using the propagator network trained only on ED volume (28) and ES volume (13), shown in green. (**B**) In the second step, the 2-nearest neighbors of the segmented frames, from the ED volume side (2, 3, 24, and 23) and from the ES volume side (17, 18, 9, and 8), shown in yellow, are segmented using the propagator network trained on ED volume (28) and its 3-nearest neighbors (29, 0, 1, 27, 26, and 25) and ES volume (13) and its 3-nearest neighbors (14, 15, 16, 12, 11, and 10), shown in green. (**C**) In the last step, the 2-nearest neighbors of the newly-segmented frames, from the ED volume side (4, 5, 22, and 21) and from the ES volume side (19, 20, 7, and 6), shown in yellow, are segmented using the propagator network trained on the rest of the frames (3, 2, 1, 0, 29, 28, 27, 26, 25, 24, 23, 8, 9, 10, 11, 12, 13, 14, 15, 16, 17, and 18), shown in green, which concludes the segmentation of all the time frames.

The label propagation procedure is explained in more detail in Algorithm 1. Firstly, for each patient, we trained a propagator network, i.e., a supervised 3D U-Net (Figure 1) using only the ground truth labels, i.e., the ED and the ES traces. Consequently, in this stage, we had only two 3D images as the training data. Subsequently, this trained propagator network was used to automatically segment adjacent 3D volumes; specifically, we chose to segment three consecutive frames directly preceding and directly following both the ED and ES frames. In this way, we segmented 12 frames using the propagator network trained on only the ED and ES volumes (Figure 2). This process resulted in 1 manually annotated ED frame, 1 manually annotated ES frame, and 12 frames with pseudo-labels. As shown in Figure 2, we pooled the original ED and ES frames with the newly segmented 12 frames and trained another propagator network using these images. This second propagator network followed the same procedure as the first, with the only exception that it segmented two consecutive frames of the training volumes. Likewise, we repeated this iterative procedure, segmenting two consecutive frames per iteration, until all the frames were segmented.

**ALGORITHM 1:**
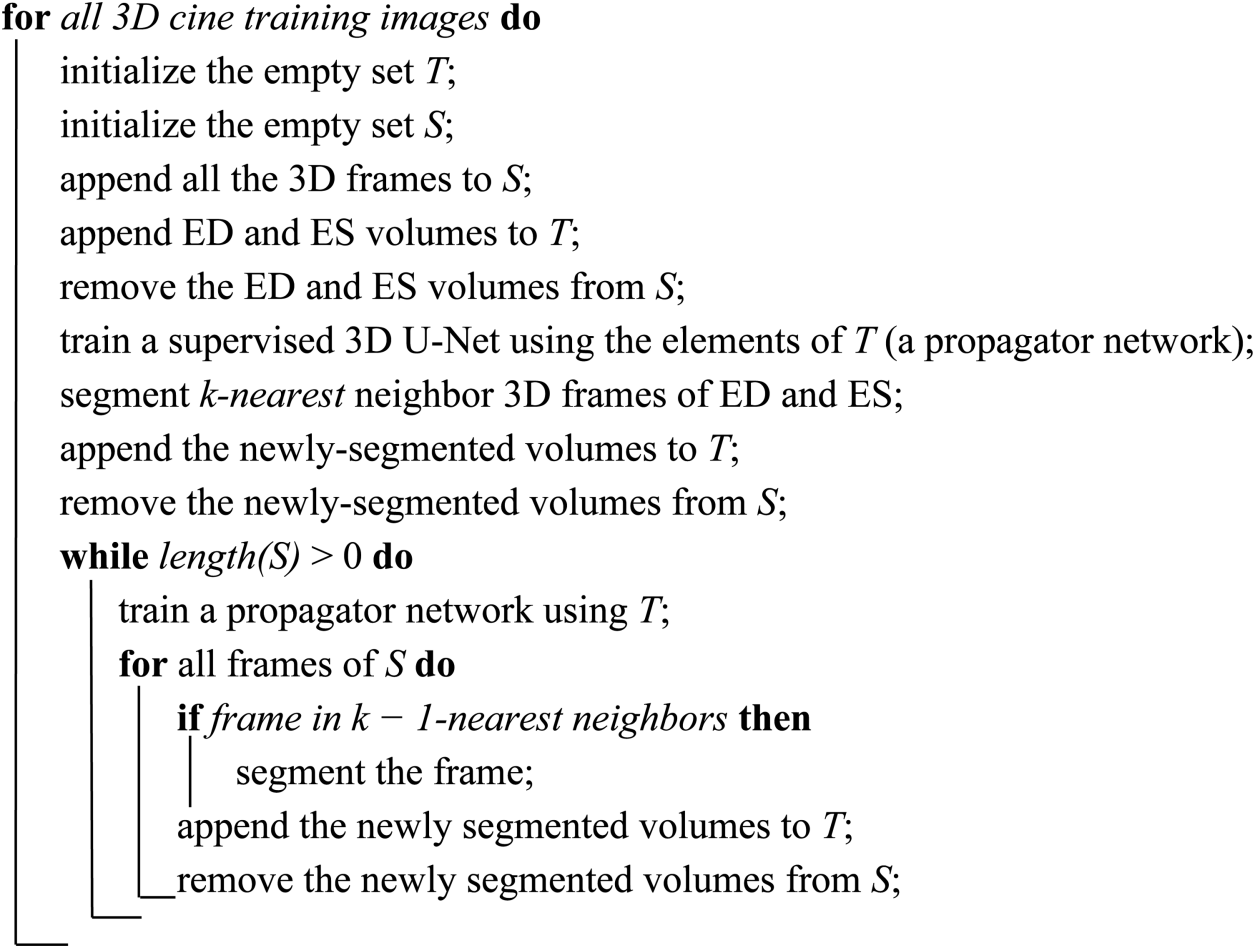
Label propagation strategy of the semi-supervised DL-based segmentation method. Here, *k* can be chosen according to the available dataset and the amount of motion between successive 3D frames of every 3D cine image. According to the 3D cine datasets and the average motion between successive 3D frames of every 3D cine image, we selected *k = 3* for all the 3D cine training images.

#### 2.3.2. Weighted-probabilistic semi-supervised training

After performing the label propagation on all the available training images and generating all the pseudo-labels for the 28 intermediate non-annotated frames corresponding to each training patient, we trained the semi-supervised DL-based segmentation method. To be consistent with the supervised training method for comparison, we used the same U-Net structure and training parameters as the supervised DL-based segmentation method with the following modifications. Firstly, the loss weights, i.e., the inverted class frequencies, were updated according to the new distribution of data for each trained 3D U-Net. Secondly, we introduced a weighted-probabilistic training process to compensate for the accumulated error during the label propagations.

To reduce error propagation during training, we performed an iteration-based probabilistic training. We assigned a different weight to each 3D frame based on its distance from the ED and ES frames. The closer the frame was to either the ES or ED frame, the greater its weight. For each 3D cine image, we divided the 30 frames into 4 sets. The ED and ES frames, which had the highest certainty of correctness for their labels, were assigned to set number 1 (*D_1_*) and called *level-1* frames. The frames that were segmented using the first 3D U-Net of the label propagation process were assigned to set number 2 (*D_2_*) and called *level-2* frames. We followed a similar strategy in dividing the remaining frames, until all 30 frames were assigned to a *D_i_* set where*, i ∈ (1,2,3,4).* Consequently, we derived a training pool ordered according to the certainty of the annotation labels. To train the network during each iteration, we randomly selected a 3D cine dataset from the 74 training datasets based on a uniform distribution. Then, we sampled two 3D volumes out of 30 frames of the chosen 3D cine dataset. The sampling occurred based on their certainty level (*D_1_*, *D_2_*, *D_3_*, and *D_4_*). The higher the level of a frame, the less its weight. Consequently, the highest weights were assigned to the D_1_ set, i.e., the ED and ES frames had higher chance of getting chosen for a training batch during each training iteration.

### 2.4. The all-frames supervised DL-based segmentation method

#### 2.4.1. Approach

To assess the effectiveness of the label propagation approach and weighted-probabilistic training, we devised a method involving a comparable 3D U-Net structure, as depicted in Figure 1. We trained this network using all the ED and ES frames from the training subjects. Afterward, we utilized the trained network to predict all unannotated frames within the training set, totaling 28 frames per training subject. The newly predicted 2,072 frames (28 × 74) were then combined with the ED and ES frames from the training subjects, resulting in a novel training set comprising 2,220 (30 × 74) 3D frames. Finally, we trained a new 3D U-Net structure using all frames from this augmented training set.

#### 2.4.2. Supervised training

We adopted a supervised learning approach to train a new 3D U-Net, as depicted in Figure 1, using all 30 frames from the 74 training datasets. Each 3D frame, whether manually annotated (ED and ES) or automatically annotated, was assigned an equal probability of selection during each training iteration. This ensured that all frames in the training datasets were treated equally during the training process.

For optimization, we utilized the AMSGrad (44) optimizer with a learning rate of *3 × 10^−4^*, along with the weighted cross-entropy loss function. The class frequencies of the training data were inverted and used as weights during training. This approach considered all the 2,220 frames in the training data to ensure an effective learning process.

### 2.5. Quantitative evaluation of the method

As our evaluation metric, we used the “Dice score” to quantify segmentation accuracy. The Dice score evaluates the overlap between automated segmentation *A* and manual segmentation *B* and is defined as,

(1) $$\mathrm{DSC}=\frac{2|A\cap B|}{\left|A|+|B\right|}$$

It is a value between 0 and 1, with 0 denoting no overlap and 1 denoting 100% overlap between the segmentation results. The higher the Dice score, the better the agreement (9). We report the Dice score values in percentage. In addition to the Dice score calculation for individual structures, we used average Dice score over the 8 cardiac structures analyzed as,

(2) $$\mathrm{DS}C_{\mathrm{total}}=\frac{1}{8}\sum_{i\in s}\mathrm{DS}C_{i},$$

where, *S* = {AO, LV, PA, RA, SVC, IVC, LA, RV}.

### 2.6. Ventricular and atrial volume measurement

We compared the ventricular and atrial volumes throughout the cardiac cycle among the manual, supervised, and semi-supervised segmentation results from the test datasets. We also reported the mean volume difference and mean volume difference expressed as a percentage over all 30 frames of the cardiac cycle.

In addition, we reformatted the 3D cine images into a short-axis view, creating a series of multiple 2D cine images that covered the ventricles from base to apex. The slice thickness of the 2D images was set at 7 mm, with no slice gap. Subsequently, the 2D cine images were manually traced to accurately outline the end-systolic and end-diastolic volumes of both the LV and RV, allowing for the calculation of ejection fractions. These calculated volumes and ejection fractions were then compared [using intraclass correlation coefficient (ICC)] to those obtained from the manual segmentation of 3D cine images and the automatic segmentation of 3D cine images using supervised and semi-supervised DL-based methods.

### 2.7. Statistical analysis of the method

Descriptive statistics are reported as median and range, or mean ± standard deviation, as appropriate. Normality was tested using Shapiro-Wilk test (46). Either a two tailed *t*-test or a Wilcoxon signed-rank test (47) were used to compare two groups of paired data with Gaussian and non-Gaussian distributions, respectively. The two tailed *t*-test was used to compare the volumetric measurements and the Wilcoxon signed-rank test was used to compare the segmentation results in terms of Dice score. A *P*-value ≤0.05 was considered statistically significant.

### 2.8. Hardware

The utilized network architecture contained merely about 1.5 million parameters enabling fast and real-time segmentation of 3D volumetric images. Consequently, the hardware we used in our experiments for training and testing the networks were Intel CPUs with 8 cores and 16 GB RAM and Nvidia GPUs of RTX 2060 with 6 GB memory.

## 3. Results

**Figure 3:**
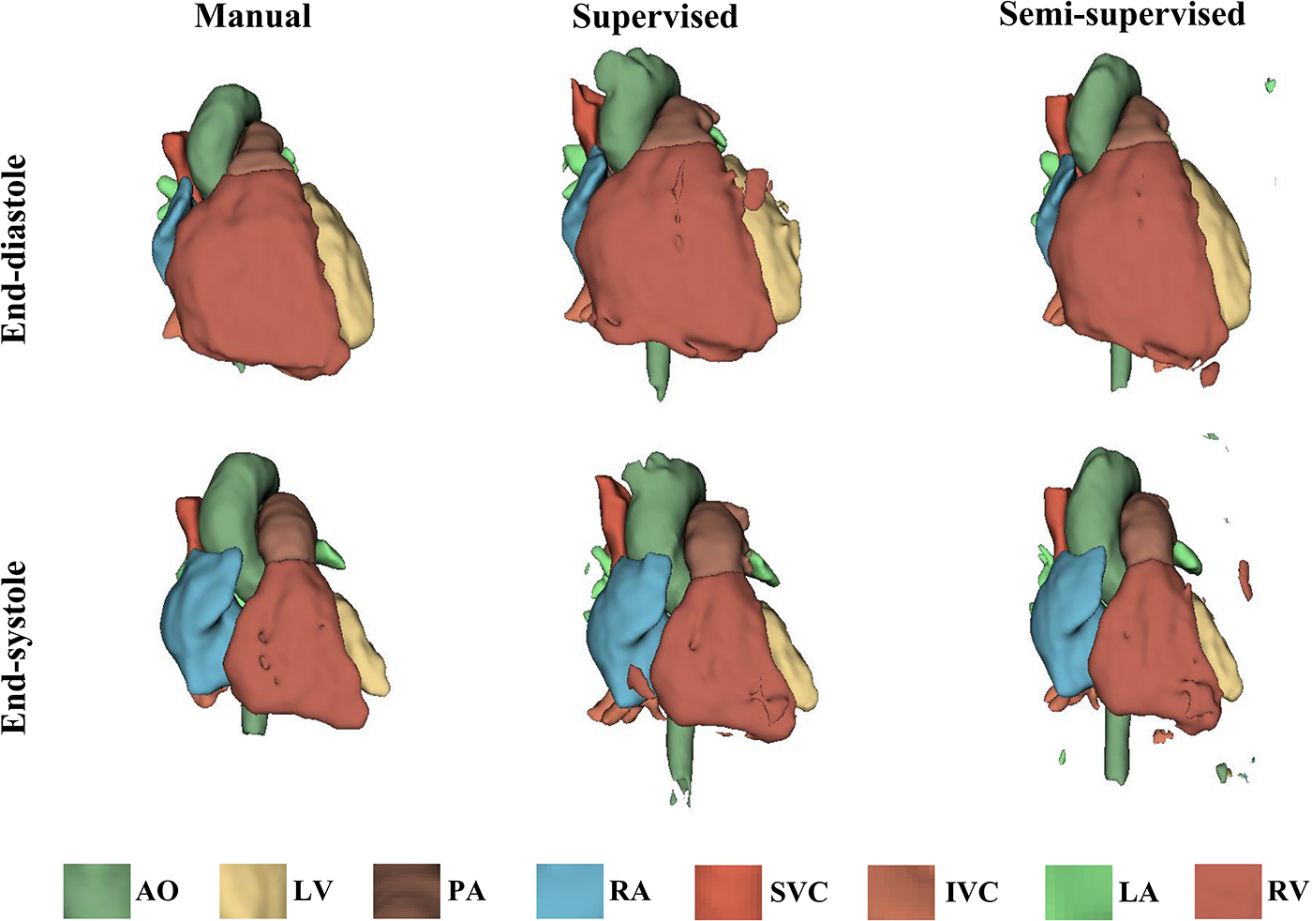
The 3D models generated from the ground truth labels (manual segmentation), the supervised, and the proposed semi-supervised DL-based segmentation methods at end-diastole and end-systole from a 17-year-old patient with arrhythmogenic right ventricular cardiomyopathy (PAT-T11 in normal category).

**Figure 4:**
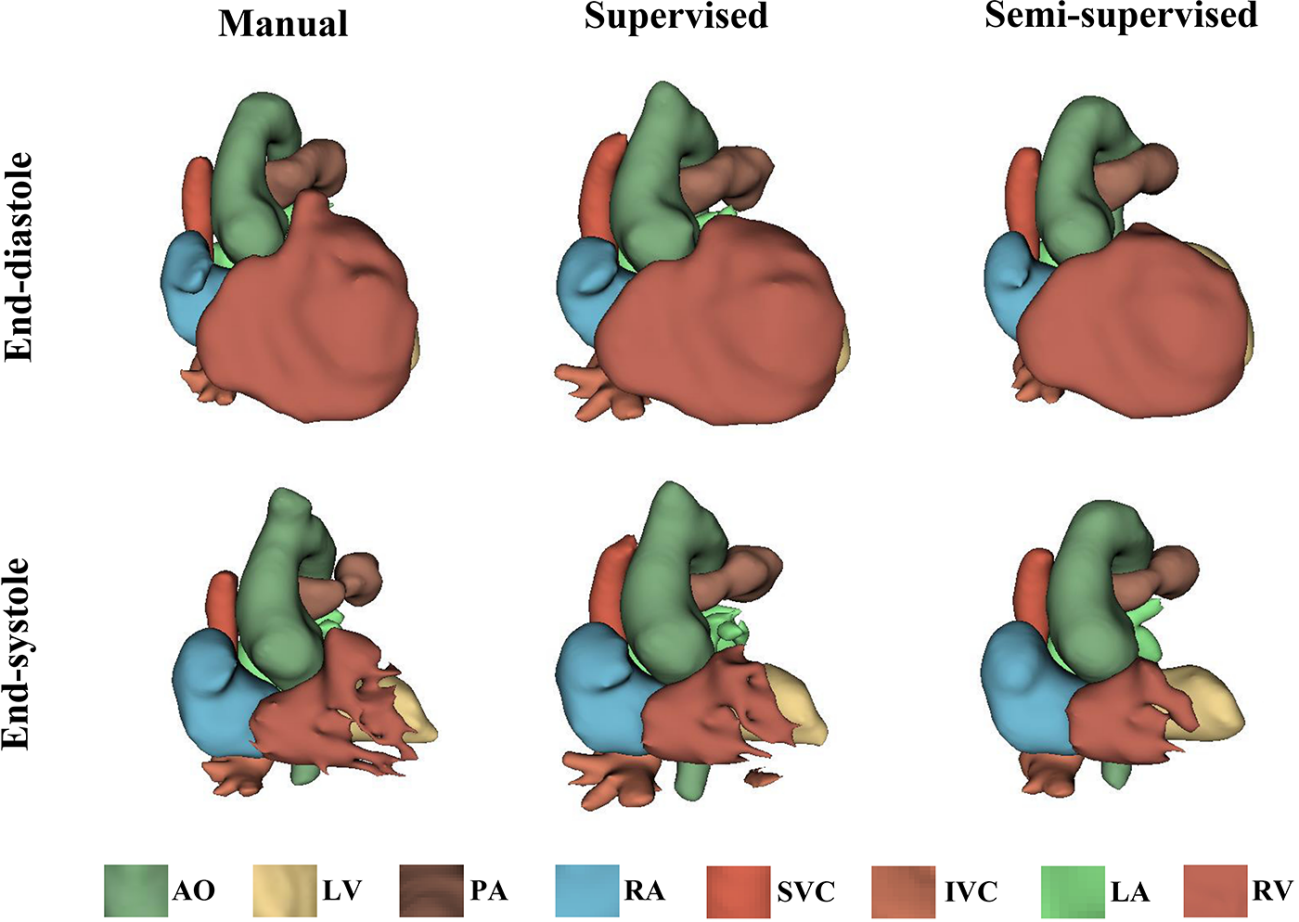
The 3D models generated from the ground truth labels (manual segmentation), the supervised, and the proposed semi-supervised DL-based segmentation method at end-diastole and end-systole from a 22-year-old patient with truncus arteriosus (PAT-T6 in mild/moderate category).

**Figure 5:**
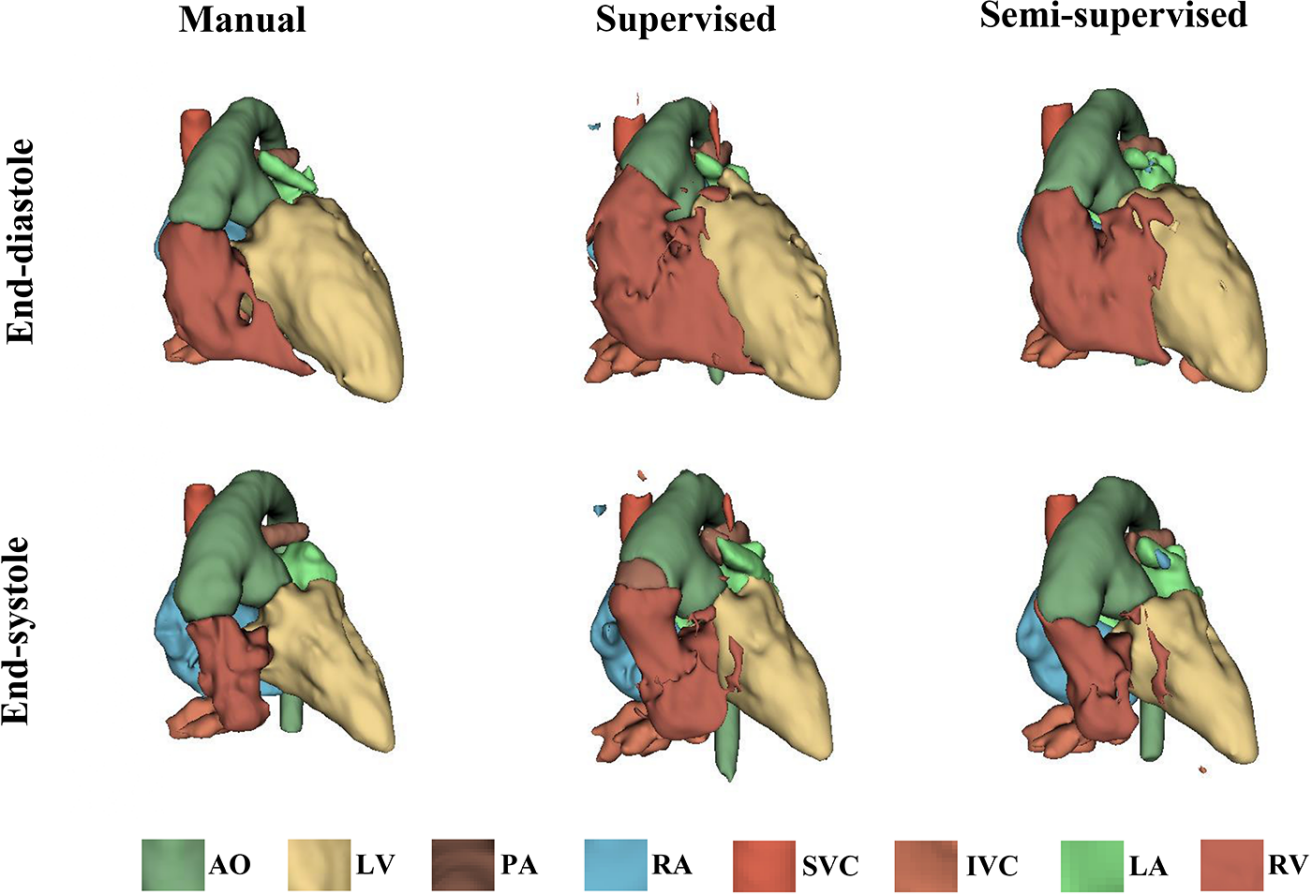
The 3D models generated from the ground truth labels (manual segmentation), the supervised, and the proposed semi-supervised DL-based segmentation method at end-diastole and end-systole cardiac frames from a 5-year-old patient with double-inlet left ventricle (PAT-T1 in severe category).

It took approximately 10 h to train the supervised DL-based segmentation method and 24 h to train the semi-supervised method. Supplementary Movie Files S1–S3 show the segmentation results of manual, supervised, and semi-supervised segmentation methods over all time frames in one test dataset with mild/moderate (subject PAT-T4 cf. Supplementary Table S2). Supplementary Movie Files S4–S6 show the dynamic 3D models of the whole-heart and great vessels from the segmentation results of manual, supervised, and semi-supervised segmentation methods over all time frames in the same test dataset. Figures 3–5, compare the ground truth labels (manual segmentation), the supervised segmentation method, and the semi-supervised segmentation method results in three patients with normal anatomy (subject PAT-T11 cf. Supplementary Table S2), mild/moderate (subject PAT-T6 cf. Supplementary Table S2), and severe (subject PAT-T1 cf. Supplementary Table S2) complexity, at ED and ES frames.

Supplementary Figure S1 shows the Dice scores for 30 frames in all the test datasets evaluating the degree of overlap between the manual and supervised DL-based segmentation methods, and manual and semi-supervised DL-based segmentation methods. The semi-supervised DL-based segmentation method had a higher average Dice score with the manual method in the segmentation of all 8 cardiac structures across all cardiac anatomy complexities, compared to the supervised DL-based segmentation method (Table 3). Supplementary Table S3 provides a comparative analysis between the proposed semi-supervised DL-based segmentation method and the all-frames supervised DL-based segmentation method. The results demonstrate that the semi-supervised DL-based segmentation method achieved significantly higher Dice scores for all structures (*P*-value ≤ 0.027) and across all three complexities (*P*-value ≤ 0.001). This indicates that the semi-supervised approach outperforms the all-frames supervised method in terms of segmentation accuracy.

**Table 3:** Statistical results comparing performance of the proposed semi-supervised segmentation method with the supervised method based on anatomic complexity category as well as structure in delineating heart chambers and great vessels in 12 3D cine test datasets.

	Dice score [%]		*P*-value
	Supervised vs. 3D manual	Semi-supervised vs. 3D manual	
Complexity			
Normal	89.71 ± 7.63	93.82 ± 4.11	**0.001**
Mild/moderate	87.52 ± 8.75	89.88 ± 6.10	**0.001**
Severe	67.71 ± 22.11	72.88 ± 20.95	**0.001**
Structure			
AO	87.19 ± 9.98	90.12 ± 6.40	**0.034**
LV	75.22 ± 31.50	79.22 ± 29.18	0.115
PA	76.88 ± 13.72	82.65 ± 12.49	**0.027**
RA	78.31 ± 26.67	83.04 ± 26.08	**0.002**
SVC	72.37 ± 15.32	83.92 ± 7.99	**0.008**
IVC	75.48 ± 9.01	78.28 ± 11.04	0.176
LA	76.84 ± 19.95	84.17 ± 11.04	**0.009**
RV	81.52 ± 15.31	84.48 ± 10.92	0.186
Over all structures	77.98 ± 19.64	83.23 ± 16.76	**0.001**

*P*-values corresponding to significant differences are indicated in bold.

**Figure 6:**
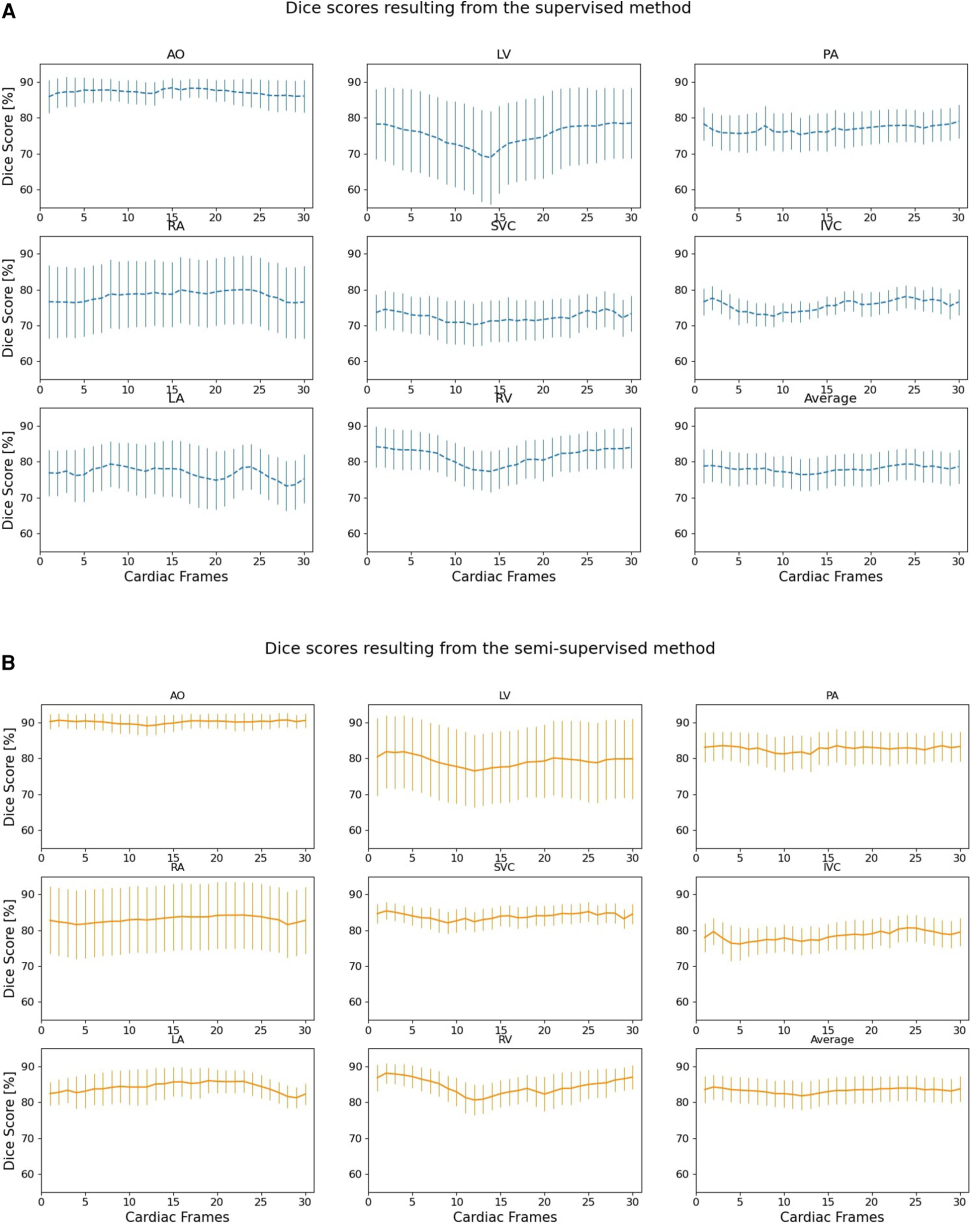
Dice scores comparing the performance of (**A**) supervised and (**B**) proposed semi-supervised DL-based segmentation methods in delineating heart chambers and great vessels throughout the cardiac cycle in 12 3D cine test datasets. Confidence intervals are shown for each graph.

Figure 6 compares the supervised and semi-supervised segmentation method in delineating different structures of the heart and great vessels over 30 frames in the cardiac cycle. As shown in Table 3, the semi-supervised method had a significantly higher Dice score compared to the supervised method for AO (*P*-value = 0.034), PA (*P*-value = 0.027), RA (*P*-value = 0.002), SVC (*P*-value = 0.008), and LA (*P*-value = 0.009); but not for LV (*P*-value = 0.115), IVC (*P*-value = 0.176), and RV (*P*-value = 0.186). On average over all structures, the performance of semi-supervised DL-based segmentation method was significantly better than that of the supervised method (*P*-value = 0.003).

**Figure 7:**
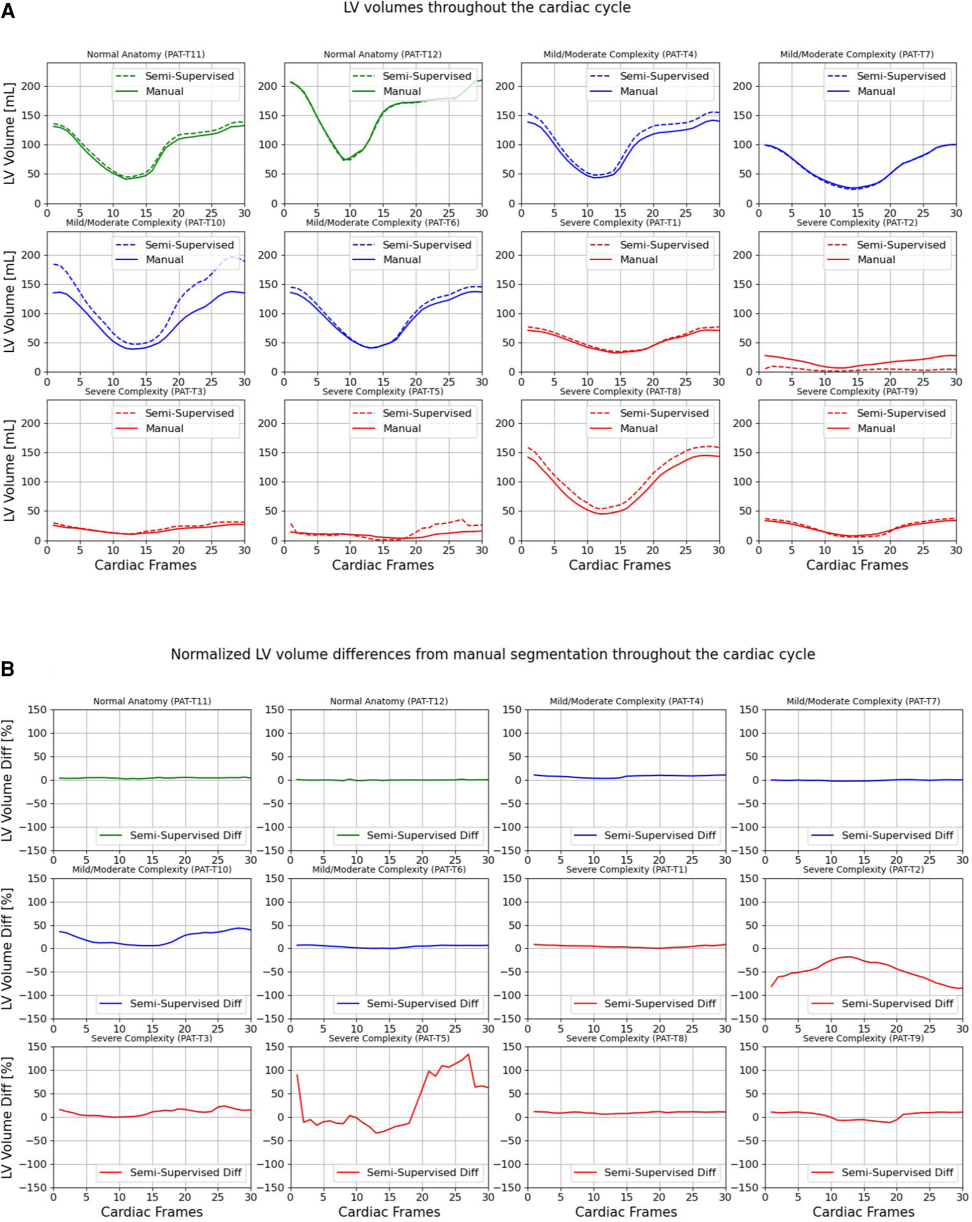
Volumes of left ventricle (LV) throughout the cardiac cycle calculated from 12 3D cine test datasets using the ground truth labels (manual segmentation) and the semi-supervised segmentation method. (**A**) Values are given in mL, representing absolute volumes. (**B**) Values are given in percentages, representing volume differences between the manual and the semi-supervised segmentation methods, normalized to the end-diastolic volume per subject.

**Figure 8:**
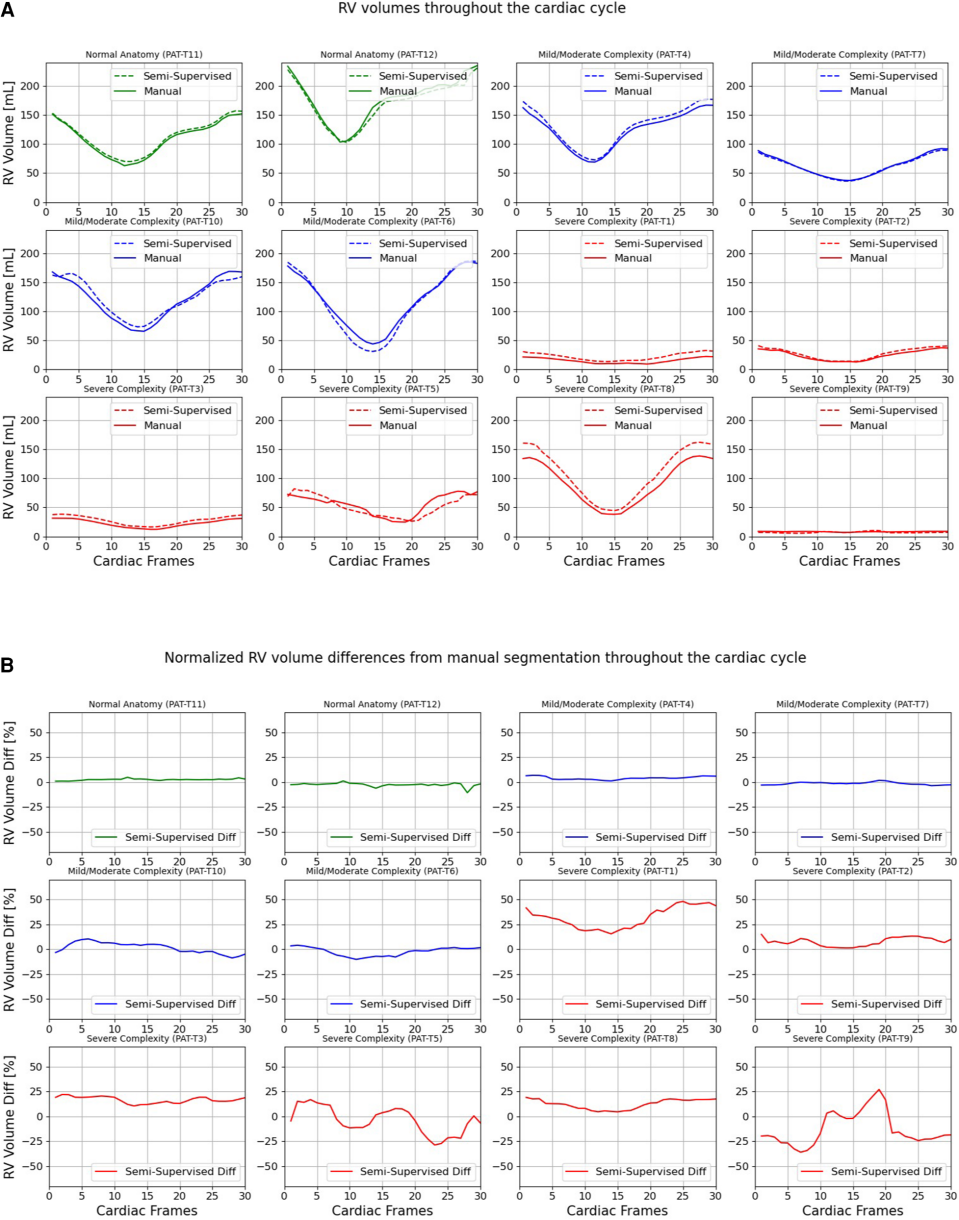
Volumes of right ventricle (RV) throughout the cardiac cycle calculated from 12 3D cine test datasets using the ground truth labels (manual segmentation) and the semi-supervised segmentation method. (**A**) Values are given in mL, representing absolute volumes. (**B**) Values are given in percentages, representing volume differences between the manual and the semi-supervised segmentation methods, normalized to the end-diastolic volume per subject.

Figures 7, 8 compare the LV and RV volumes computed from the manual segmentation and semi-supervised segmentation method in the test datasets. Similarly, Supplementary Figures S2, S3 compare the LV and RV volumes computed from the manual segmentation and supervised segmentation method in the test datasets. As shown in Table 4, the overall mean volume difference error between the manual and semi-supervised segmentation was less than the mean difference error between the manual and supervised segmentation for LV and RV.

**Table 4:** Statistical results comparing LV, RV, LA, and RA volumes computed from the manual segmentation and supervised and semi-supervised segmentation methods in 12 3D cine test datasets using all 30 frames of the cardiac cycles.

Structure	Supervised vs. 3D manual			Semi-supervised vs. 3D manual		
	Difference [ml]	Difference [%]	*P*-value	Difference [ml]	Difference [%]	*P*-value
LV	10.0 ± 15.0	21.2 ± 76.8	0.030	5.2 ± 11.8	4.0 ± 40.8	**0.114**
RV	8.1 ± 11.1	24.1 ± 38.7	0.017	2.4 ± 8.1	7.1 ± 18.9	**0.212**
LA	3.9 ± 9.0	1.9 ± 32.6	**0.137**	1.9 ± 5.1	4.9 ± 16.3	**0.205**
RA	10.1 ± 10.2	32.5 ± 52.1	0.003	4.2 ± 4.0	13.1 ± 19.5	0.003

*P*-values corresponding to significant differences are indicated in bold.

**Figure 9:**
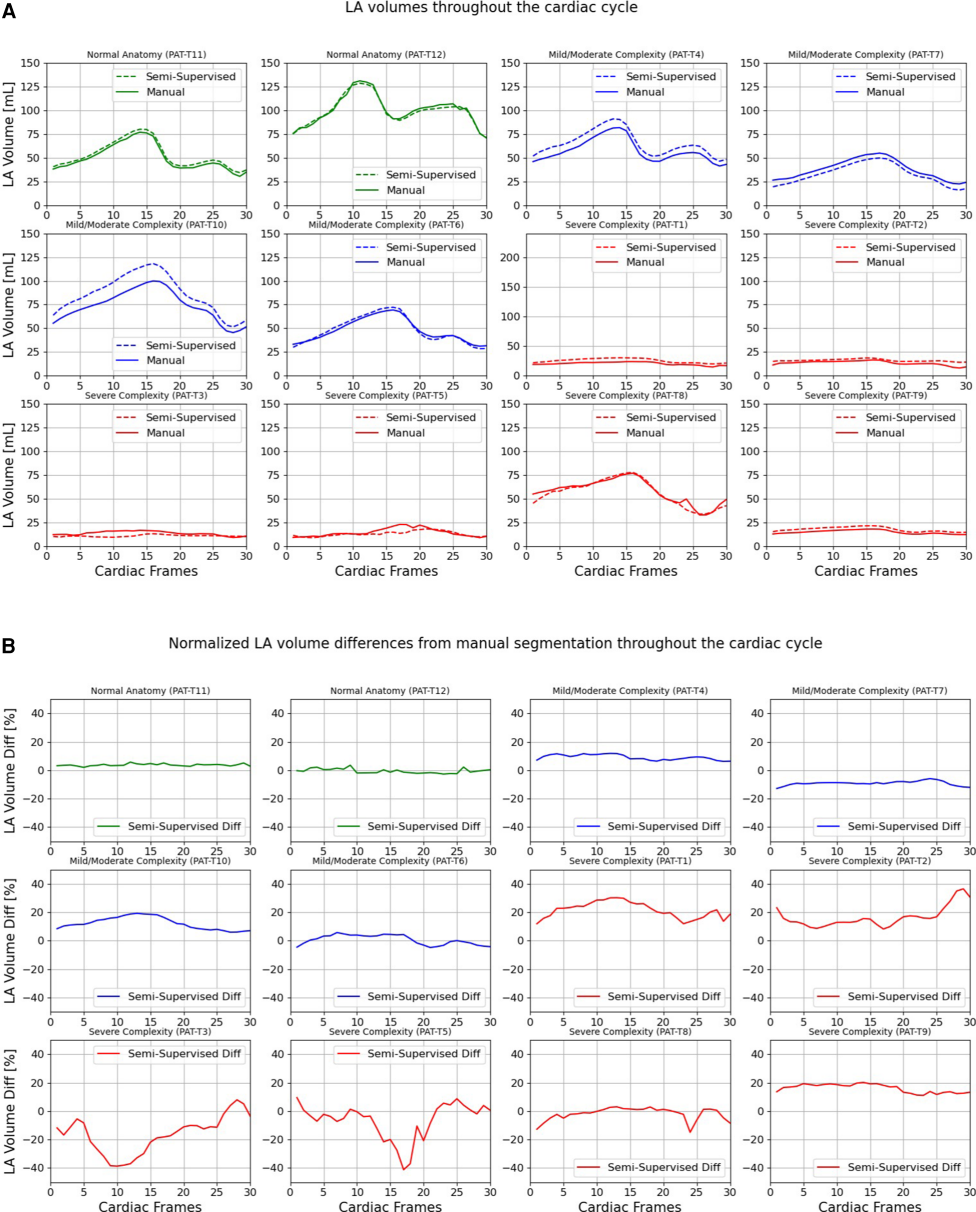
Volumes of left atrium (LA) throughout the cardiac cycle calculated from 12 3D cine test datasets using the ground truth labels (manual segmentation) and the semi-supervised segmentation method. (**A**) Values are given in mL, representing absolute volumes. (**B**) Values are given in percentages, representing volume differences between the manual and the semi-supervised segmentation methods, normalized to the largest atrial volume per subject.

**Figure 10:**
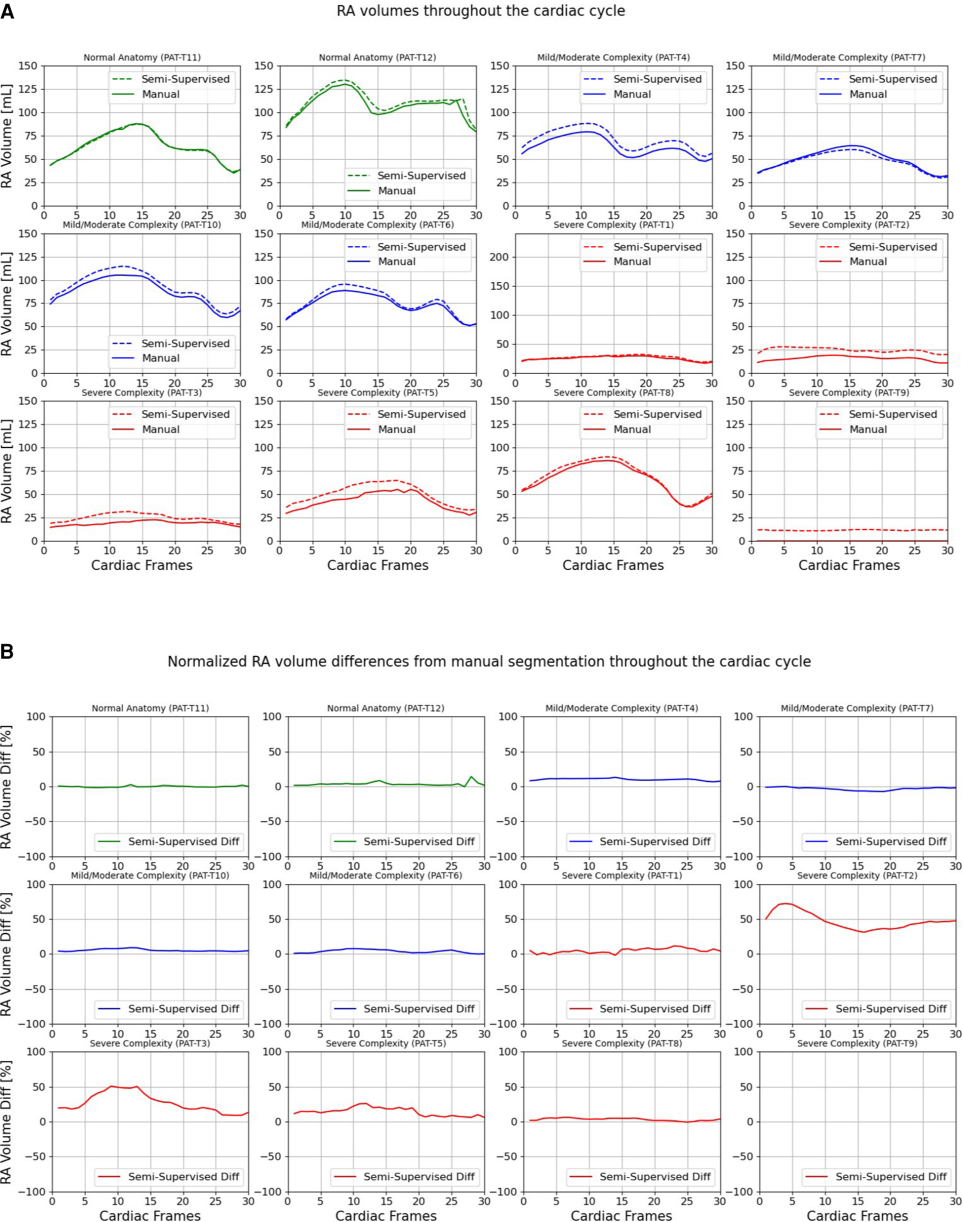
Volumes of right atrium (RA) throughout the cardiac cycle calculated from 12 3D cine test datasets using the ground truth labels (manual segmentation) and the semi-supervised segmentation method. (**A**) Values are given in mL, representing absolute volumes. (**B**) Values are given in percentages, representing volume differences between the manual and the semi-supervised segmentation methods, normalized to the largest atrial volume per subject. PAT-T9 refers to a Fontan patient without RA.

Figures 9, 10 compare the LA and RA volumes between the manual segmentation and semi-supervised segmentation method, and Supplementary Figures S4, S5 compare the LA and RA volumes between the manual segmentation and supervised segmentation method in the test datasets. As shown in Table 4, the overall mean volume difference error between the manual and semi-supervised segmentation was less than the mean difference error between the manual and the supervised segmentation for LA and RA.

Table 5 presents a comparison of ventricular volumes and ejection fractions obtained through various segmentation methods, including manual segmentation of reformatted 2D cine images, manual segmentation of 3D cine images, and both automatic supervised and semi-supervised DL-based segmentation methods. The results demonstrate a strong agreement between ventricular volumes and ejection fractions obtained from the manual segmentation of 3D cine images and the automatic segmentation of 3D cine images using a semi-supervised DL-based algorithm (*p*-value > 0.087; ICC > 0.94). The manual 3D segmentation also exhibited good agreement with manual 2D segmentation in measuring ventricular volumes and ejection fractions (ICC > 0.96). However, the end-diastolic volumes of the LV and RV, calculated from manual segmentation of 3D cine images, were significantly larger than those measured from manual segmentation of reformatted 2D images (*P*-value < 0.05). Consequently, this difference resulted in distinct ejection fractions when comparing manual segmentation of 3D cine and reformatted 2D cine images (*P*-value < 0.05).

**Table 5:** Statistical results comparing ventricular volumes computed from the manual segmentation of reconstructed 2D cine images with the manual segmentation of 3D cine images and automatic segmentation of 3D cine images using supervised and semi-supervised methods in 12 3D cine test datasets.

	Left ventricle (LV)			Right ventricle (RV)		
	End-diastole [ml]	End-systole [ml]	Ejection fraction [%]	End-diastole [ml]	End-systole [ml]	Ejection fraction [%]
Manual (2D)	86.4 ± 51.9	31.8 ± 18.6	62.4 ± 5.6	87.0 ± 55.1	42.1 ± 27.5	47.4 ± 15.7
Manual (3D)	95.1 ± 55.1	32.0 ± 19.9	66.4 ± 7.4	106.5 ± 68.2	43.5 ± 31.8	56.4 ± 14.0
Supervised (3D)	109.2 ± 64.7	35.7 ± 26.0	71.2 ± 9.5	115.9 ± 72.6	48.8 ± 30.2	51.2 ± 15.6
Semi-supervised (3D)	104.7 ± 63.7	33.9 ± 22.7	68.4 ± 7.9	108.8 ± 67.2	43.4 ± 30.4	55.0 ± 18.3

## 5. Discussion

We developed automatic supervised and semi-supervised DL-based segmentation methods for delineating great vessels (AO, PA, SVC, and IVC) and cardiac chambers (LV, RV, LA, and RA) from 3D cine whole-heart CMR images acquired from patients with a wide range of CHD lesions, based on a 3D U-Net structure (37, 38) and benefiting from the nnU-Net (41) configurations, one of the state-of-the-art frameworks for volumetric segmentation. We divided the patients into 3 categories based on complexity of cardiac anatomical variations (normal anatomy, and mild**/**moderate and severely complex) and investigated the performance of the automatic DL-based segmentation methods for each group. The semi-supervised segmentation method performed significantly better than the supervised method in each complexity category. However, the performance of the supervised and semi-supervised segmentation methods significantly decreased as anatomical complexity increased.

On average, the semi-supervised DL-based segmentation method outperformed the supervised DL-based segmentation method in delineating the great vessels and heart chambers. This likely stems from the fact that the semi-supervised segmentation method was trained on all 30 phases in the cardiac cycle while the supervised method was trained only on the ES and ED cardiac phases. In order to further explore the implications of training on all 30 frames, we compared our proposed semi-supervised DL-based segmentation method against the all-frames supervised DL-based segmentation method. The all-frames supervised DL-based segmentation method was also trained on all 30 phases without incorporating our novel label propagation technique. As anticipated, the semi-supervised DL-based segmentation method demonstrated significantly superior segmentation results, thus validating its effectiveness. This outcome could potentially be attributed to the fact that errors would accumulate more significantly in the all-frames supervised DL-based segmentation method for frames that are further away from the ED and ES frames, i.e., the frames containing manual annotations.

In subjects where there was only one ventricle, the network had difficulties determining whether the single ventricle was anatomically left or right, leading to a decrease in Dice scores for either the LV or RV. Consequently, the fact that the LV and RV Dice scores were not significantly different between the supervised and semi-supervised methods (Table 4) could be due to lower Dice scores for the aforementioned reason. In terms of volume measurements, there were no significant differences between the volumes of LV, RV, or LA calculated from manual segmentation (reference) and semi-supervised segmentation method. However, the volumes measured by the supervised method were significantly different compared to manual segmentation, except for the LA. This could be due to less dynamic motion of LA between ED and ES frames compared to the ventricles so that the supervised method (which was trained only on ED and ES frames) could perform as well as the semi-supervised method (which was trained on all 30 frames).

Like ours, previously described DL-based segmentation approaches can perform automatic delineation of great vessels and heart chambers. Pace et al. (25, 39) investigated the potential of active learning to automatically solicit user input in areas where segmentation error is likely to be high with an interactive algorithm. The objective was to segment the great vessels and heart chambers from static whole-heart 3D CMR images in patients with CHD. However, their algorithm was trained on one cardiac phase (i.e., ED phase). Since there is relatively large cardiac motion from ED to ES frames, it is not clear how their segmentation method would perform throughout the cardiac cycle. Qin et al. (24) presented a DL-based segmentation method for combined motion estimation and delineation of heart chambers from CMR images. However, their method is developed for 2D (not 3D) cine images. Bai et al. (48) proposed a CMR image segmentation method which combines fully convolutional and recurrent neural networks. They utilized a nonrigid registration algorithm for label propagation that accounts for motion of the heart in all cardiac frames. However, their method utilized a non-rigid registration algorithm for label propagation as opposed to our DL-based label propagation method. The performance of DL-based label propagation method needs to be compared with non-rigid label propagation methods. Furthermore, their method was developed for segmenting only AO. Recently, Cao et al. (30), Liu et al. (31), Hatamizadeh et al. (32), and Chen et al. (33) have proposed further general-purpose vision-transformer-based [Dosovitskiy et al. (36)] network architectures for 3D medical image segmentation. However, vision transformer architectures are known for their high computational resource requirements which would not suit our iterative algorithm (49). In addition, their performances have not yet been validated on the segmentation of CMR images.

Our study has several limitations. A large number of patients had a coronary sinus close to their RA and it was not included in our segmentation labels due to its small size, significant motion, and difficulty in identification and manual segmentation. Furthermore, pulmonary veins were included in the segmentation of LA. Consequently, future work could consider the coronary sinus and pulmonary veins as additional structures that may require manual segmentation for training DL-based segmentation methods. In our manual segmentation of AO, we allowed the descending AO to have different lengths for each patient, as determined by the DL-based segmentation method. Similarly, SVC and IVC have different lengths for each patient in our manual segmentation, and for patients with an interrupted IVC, the hepatic veins were often segmented as IVC. We let the DL-based segmentation methods decide where the starting point of SVC and IVC would be. Hence, the lengths of AO, SVC, and IVC were different between the results of manual and automatic DL-based segmentation methods causing slightly lower Dice scores for the AO, SVC, and IVC. In addition, we did not have a uniform number of subjects assigned to each complexity category in the test. The test and training datasets could have been better balanced across complexity categories by increasing the number of patients. Furthermore, the number of test subjects (*n* = 12) could be increased. However, a major constraint in preparation of the data was the fact that manual annotation of the 3D cine datasets was extremely time-consuming, given that for each 3D cine image in the test dataset, all the 30 frames needed to be manually segmented. Cross-validation could be helpful in this regard; however, this would require all 30 frames of training and validation images to be manually segmented, which was infeasible. Future work will consider increasing the number of manually segmented datasets for training, validation, and test. The performance of recurrent neural networks (RNN) (50–53) in automatically segmenting the 30 time frames of cardiac cycle in the 3D cine datasets should also be evaluated and compared with the performance of proposed supervised and semi-supervised DL-based segmentation methods.

Our study demonstrated the accurate segmentation of ventricular volumes using the automatic semi-supervised DL-based segmentation method compared to the manual segmentation of 3D cine images. However, a bias was observed between the manual segmentation of 3D cine images and reformatted 2D cine images when calculating the end-diastolic volumes of LV and RV. These biases, approximately 9 ml for LV and 19 ml for RV, may be attributed to the challenge of accurately delineating the atrial and ventricular cavity in the basal region during the manual segmentation of reformatted short-axis 2D cine images. In our future study, we plan to investigate the underlying reasons for this bias between manual 3D segmentation and manual segmentation of reformatted 2D images.

Finally, our DL-based segmentation methods did not take advantage of known patient diagnoses, which could provide significant information about the locations of great vessels and heart chambers. For instance, in cases where there was only one atrium (subject PAT-T9 cf. Supplementary Table S2), the networks had difficulty assigning left or right descriptors to the atrium present, e.g., subject PAT-T9 cf. Supplementary Table S2 did not have RA. The absence of RA had affected the neighbouring structure, i.e., RV and the network assigned a very small part of the RV to RA as well. Many cases of single ventricle, dextrocardia, and common atrium resulted in the networks mistakenly assigning LV to RV or LA to RA, leading to a significant decrease in Dice score. For subject PAT-T2 cf. Supplementary Table S2, which had inverted ventricles, although the network was able to segment the ventricles almost perfectly, it had difficulties in differentiating between LV and RV and assigned a part of the LV to RV. In Fontan patients (subject PAT-T9 cf. Supplementary Table S2), the networks struggled to differentiate between the IVC and Fontan conduits, resulting in a low Dice score. Similarly, the networks failed to accurately identify where to stop the SVC in Glenn patients (subject PAT-T2 cf. Supplementary Table S2). By incorporating diagnosis information into the training and decision-making phases of the networks, the networks’ performance in correctly assigning segment labels could be significantly improved.

Supervised and semi-supervised DL-based segmentation methods were presented for delineating the whole-heart and great vessels from 3D cine CMR images of patients with different types of CHD. The performance of the semi-supervised method was shown to be better than the supervised segmentation technique. The semi-supervised DL-based segmentation method accurately delineates the heart chambers and great vessels and can be used to calculate ventricular and atrial volumes throughout the cardiac cycle. Such a segmentation technique can reduce inter- and intra-observer variability and make CMR exams more standardized and efficient.

## Data Availability

The datasets presented in this article are not readily available because the datasets used and/or analyzed during the current study are internal data of Boston Children's Hospital and are available from the corresponding authors on reasonable request. Requests to access the datasets should be directed to AP, andrew.powell@cardio.chboston.org.
