# Supplementary material for: Automated segmentation of 3D cine cardiovascular magnetic resonance imaging
Source: Front Cardiovasc Med. 2023 Oct 13;10:1167500. doi: 10.3389/fcvm.2023.1167500 (PMC10613522; doi:10.3389/fcvm.2023.1167500)
Supplement: Supplementary file 8 [file Datasheet1.pdf]

Supplementary Material

1 SUPPLEMENTARY TABLES AND FIGURES

Supplementary Table S1. Distribution of patients in the 3D cine datasets based on anatomic complexity category.

Subset	Normal	Mild/Moderate	Severe	Total
**Training**	20	29	25	74
**Validation**	4	6	3	13
**Test**	2	4	6	12
**Sum**	26	39	34	99

Supplementary Table S2. Qualitative assessment for each of the 12 3D cine test datasets. The Dice score corresponds to the average over all structures and frames for each patient, obtained using the semi-supervised DL-based segmentation method.

Test Datasets	Pathology	Complexity	Dice Score [%]	Qualitative Assessment
**PAT-T1**	Double-inlet left ventricle, transposition of the great arteries, S/P bidirectional Glenn and Damus-Kaye-Stansel anastomosis	Severe	85.8 ± 5.2	At the ventricular septal defect, some of the left ventricle is segmented as right ventricle. At the atrial septal defect some of the left atrium is segmented as right atrium
**PAT-T2**	Dextrocardia with apex pointing anterior/midline. L-loop transposition of the great arteries, ventricular septal defect, S/P bidirectional Glenn	Severe	75.0 ± 16.7	The right atrium segmentation extends into both the left ventricle and left atrium and borders both ventricles. The left atrium does not border either ventricle. A significant portion of the left ventricle is not segmented at all. At end systole, even less of the left ventricle blood pool is segmented as left ventricle; there is still a large portion of unsegmented left ventricle blood pool, and now some of the right ventricle segmentation extends into the left ventricle. Same appearance of right atrium segment extending into left ventricle and left atrium
**PAT-T3**	Dextrocardia, heterotaxy syndrome, complete atrioventricular canal, pulmonary atresia	Severe	75.6 ± 11.7	The left-sided atrium was labeled right atrium likely because it is in communication with the right ventricle. The right-sided atrium was labeled as left atrium and erroneously covered part of the left-sided atrium where the pulmonary veins entered. The left ventricle segment includes some of the right-sided atrium. The plane of the ventricular septum at the ventricular septal defect was a little off to favor a larger left ventricle. In end-systole, the portion of the left-sided atrium that was segmented as left ventricle at diastole is now segmented as right atrium. The pulmonary venous entrance into the right-sided atrium is labeled as left

				atrium and the remainder of both atria are labeled right atrium
**PAT-T4**	Atrial septal defect S/P surgical closure	Mild/Moderate	91.4 ± 3.3	No errors
**PAT-T5**	Superior-inferior ventricles, double-outlet right ventricle, pulmonary stenosis, straddling mitral valve, conoventricular septal defect, S/P patent ductus arteriosus stent and subsequent bidirectional Glenn and atrial septectomy	Severe	66.2 ± 26.7	The entire left ventricle is segmented as the right ventricle. Most of the right ventricle is segmented as right ventricle but has a small component of left ventricle segmentation within it. The positioning of the ventricles is not only inverted, but the plane of the ventricular septum is in an abnormal orientation making segmentation difficult. A portion of the proximal thoracic descending aorta and proximal left pulmonary artery are not segmented due to metal artifact (patent ductus arteriosus stent). The pulmonary root location is in the typical location of an aortic root and the left atrium and aortic segmentation both cover some of the pulmonary root
**PAT-T6**	Truncus arteriosus, anomalous return of an accessory right upper lobe pulmonary vein to the superior vena cava, S/P truncus repair with right-ventricle-to-pulmonary-artery conduit & patch closure of ventricular septal defect, S/P placement of a stent and Melody valve in the conduit	Mild/Moderate	88.4 ± 5.8	The right ventricle to pulmonary artery conduit is not visible or segmented due to metal artifact relating to a Melody valve inside the conduit. At end systole, some of the metal artifact and unsegmented area extends down further into the right ventricle outflow tract
**PAT-T7**	Congenital aortic stenosis, S/P neonatal balloon aortic valvuloplasty and subsequent surgical aortic valvuloplasty	Mild/Moderate	91.2 ± 8.9	No errors
**PAT-T8**	Tetralogy of Fallot with pulmonary atresia; S/P ventricular septal defect closure and right-ventricle-to-pulmonary-artery conduit; S/P multiple conduit revisions and patch closure of coronary sinus septal defect	Mild/Moderate	85.5 ± 8.1	Intracardiac and vascular segmentation is correct. Some epicardial fat versus pericardial fluid is segmented by multiple labels
**PAT-T9**	Single ventricle with dominant left ventricle and hypoplastic right ventricle, S/P Fontan procedure	Severe	63.5 ± 27.4	The Fontan conduit was labeled as right atrium. Patient is s/p Damus-Kaye-Stansel anastomosis and native aortic root is labeled as aorta and native pulmonary root is labeled as pulmonary artery inferior to the anastomosis
**PAT-T10**	Ventricular septal defect repair, S/P surgical closure and subsequent aortic valve replacement	Mild/Moderate	88.5 ± 4.2	The lateral basal portion of the right ventricle is labeled as left ventricle. This area is in continuity with the metal artifact from the prosthetic aortic valve. The prosthetic aortic valve is obscured by artifact and this portion is not segmented
**PAT-T11**	MRI to rule out arrhythmogenic cardiomyopathy	Normal	93.4 ± 4.3	A portion of stomach lumen was segmented as hepatic vein
**PAT-T12**	Marfan syndrome	Normal	94.2 ± 3.9	Small, isolated islands of segmentation below the diaphragm

Supplementary Table S3. Statistical comparison of the semi-supervised DL-based segmentation method with the all-frames supervised DL-based segmentation method. Results are sorted according to anatomical complexity category and structure, focusing on the delineation of heart chambers and great vessels across 12 3D cine test datasets.

	**Dice score [%]**		**P-value**
	**All-frames supervised**	**Semi-supervised**	
**Complexity**			
**Normal**	84.96 ± 8.70	93.82 ± 4.11	**0.001**
**Mild/Moderate**	83.77 ± 8.45	89.88 ± 6.10	**0.001**
**Severe**	65.06 ± 21.28	72.88 ± 20.95	**0.001**
**Structure**			
**AO**	86.60 ± 6.01	90.12 ± 6.40	**0.002**
**LV**	68.38 ± 30.52	79.22 ± 29.18	**0.016**
**PA**	73.06 ± 12.87	82.65 ± 12.49	**0.021**
**RA**	76.81 ± 26.45	83.04 ± 26.08	**0.001**
**SVC**	69.89 ± 13.16	83.92 ± 7.99	**0.005**
**IVC**	68.90 ± 9.01	78.28 ± 11.04	**0.027**
**LA**	76.16 ± 14.04	84.17 ± 11.04	**0.001**
**RV**	77.11 ± 17.19	84.48 ± 10.92	**0.023**
**Over all structures**	74.61 ± 18.82	83.23 ± 16.76	**0.001**

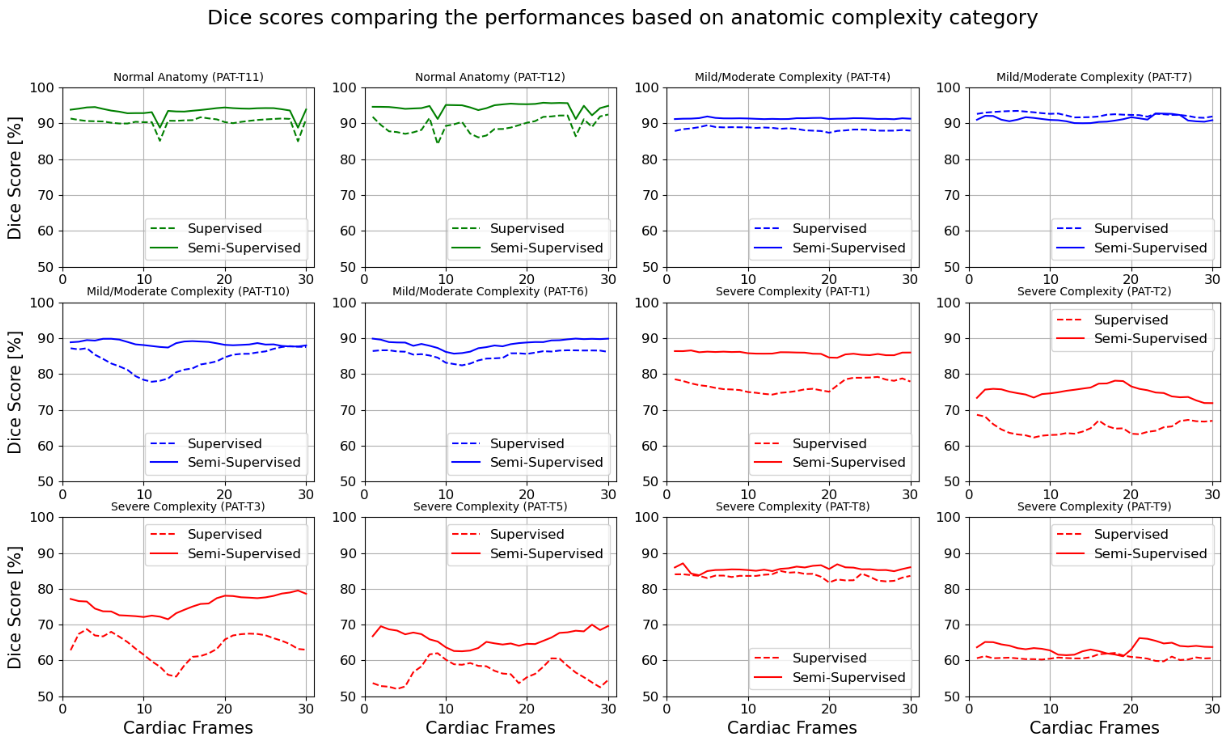

Supplementary Figure S1. Dice scores comparing the performance of the supervised and the proposed semi supervised DL-based segmentation methods in delineating heart chambers and great vessels throughout the cardiac cycle in 12 3D datasets, based on anatomic complexity category.

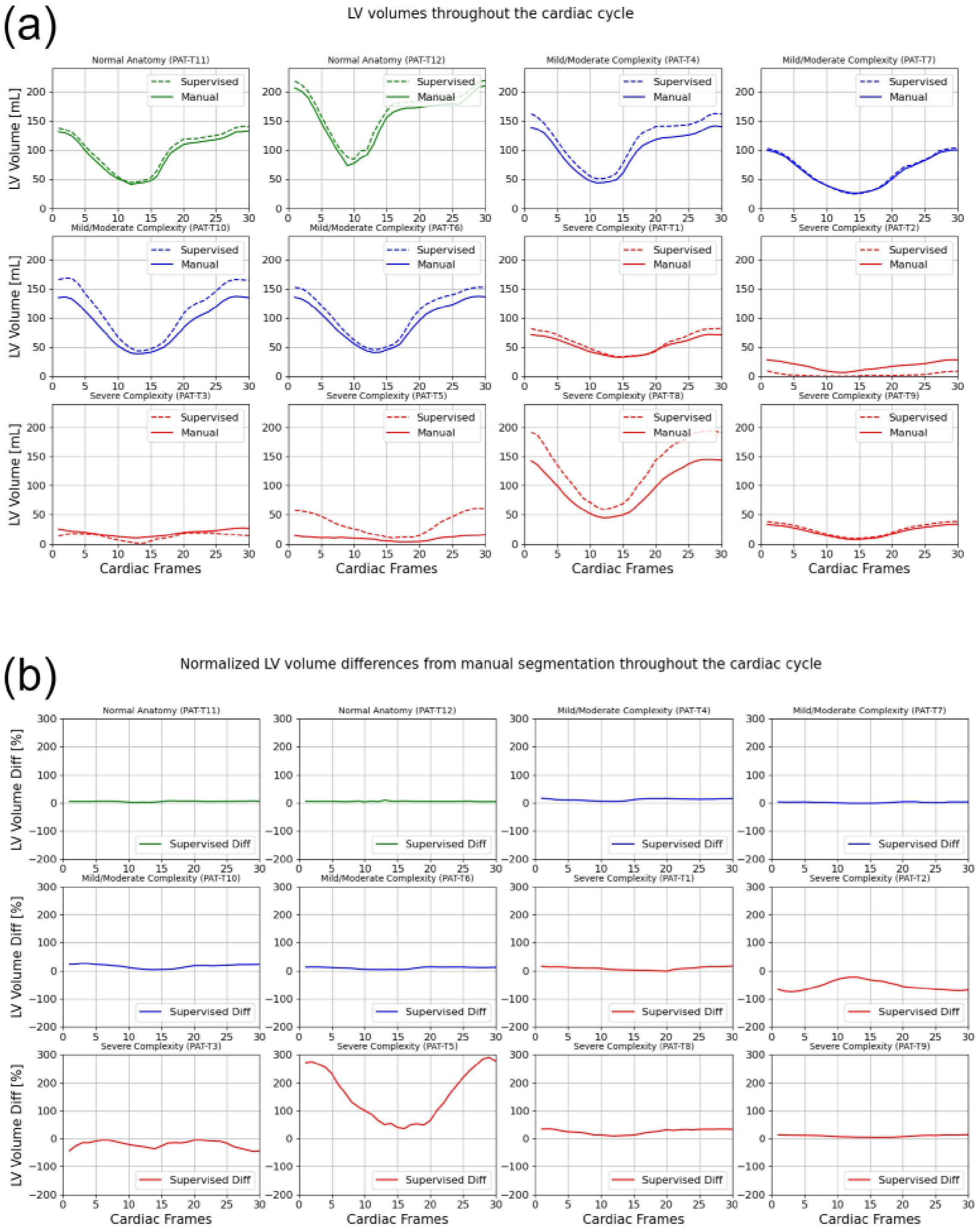

Supplementary Figure S2. Volumes of left ventricle (LV) throughout the cardiac cycle calculated from 12 3D cine test datasets using the ground truth labels (manual segmentation) and the supervised segmentation method. (a) Values are given in mL, representing absolute volumes. (b) Values are given in percentages, representing volume differences between the manual and the supervised segmentation methods, normalized to the end-diastolic volume per subject.

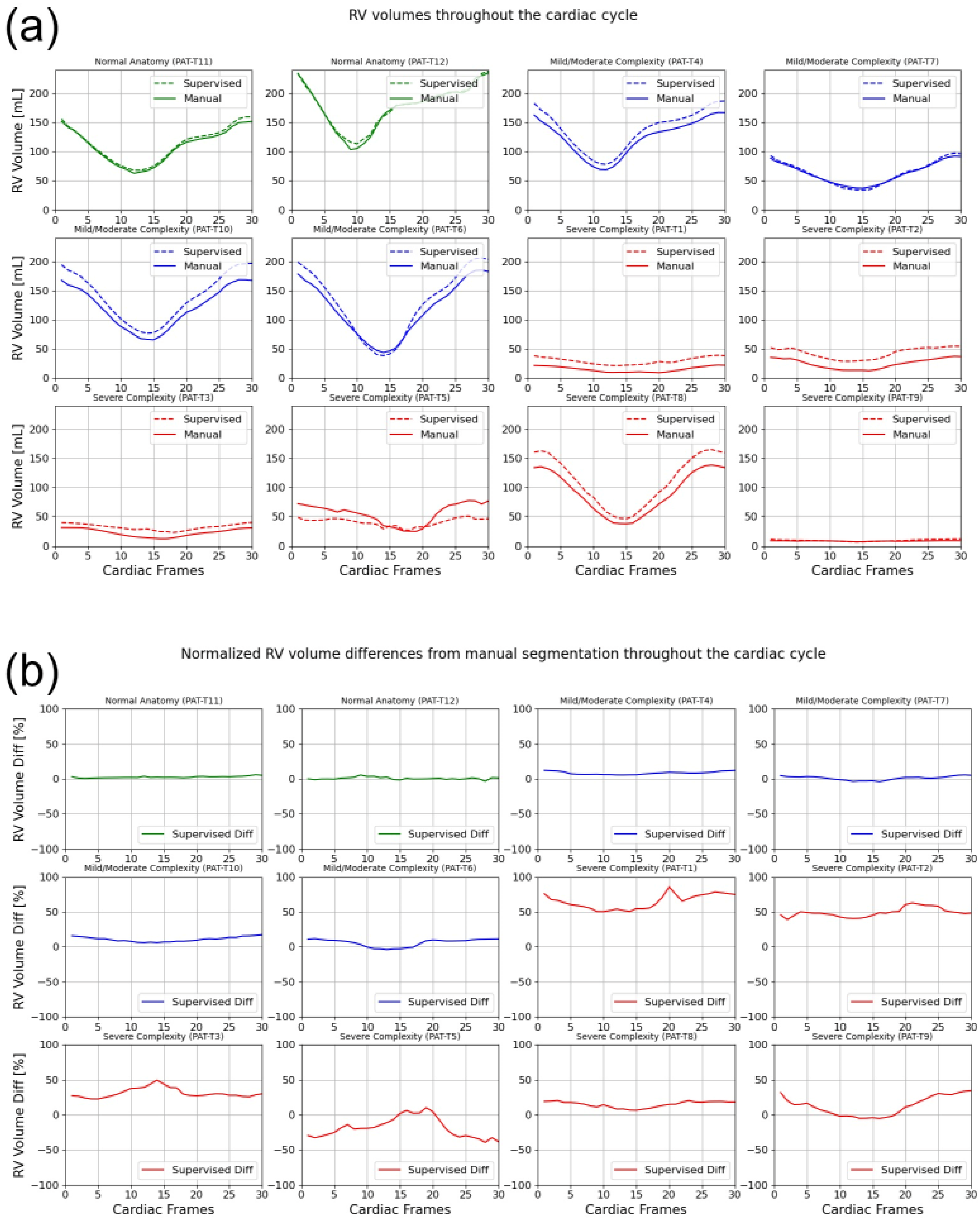

Supplementary Figure S3. Volumes of right ventricle (RV) throughout the cardiac cycle calculated from 12 3D cine test datasets using the ground truth labels (manual segmentation) and the supervised segmentation method. (a) Values are given in mL, representing absolute volumes. (b) Values are given in percentages, representing volume differences between the manual and the supervised segmentation methods, normalized to the end-diastolic volume per subject.

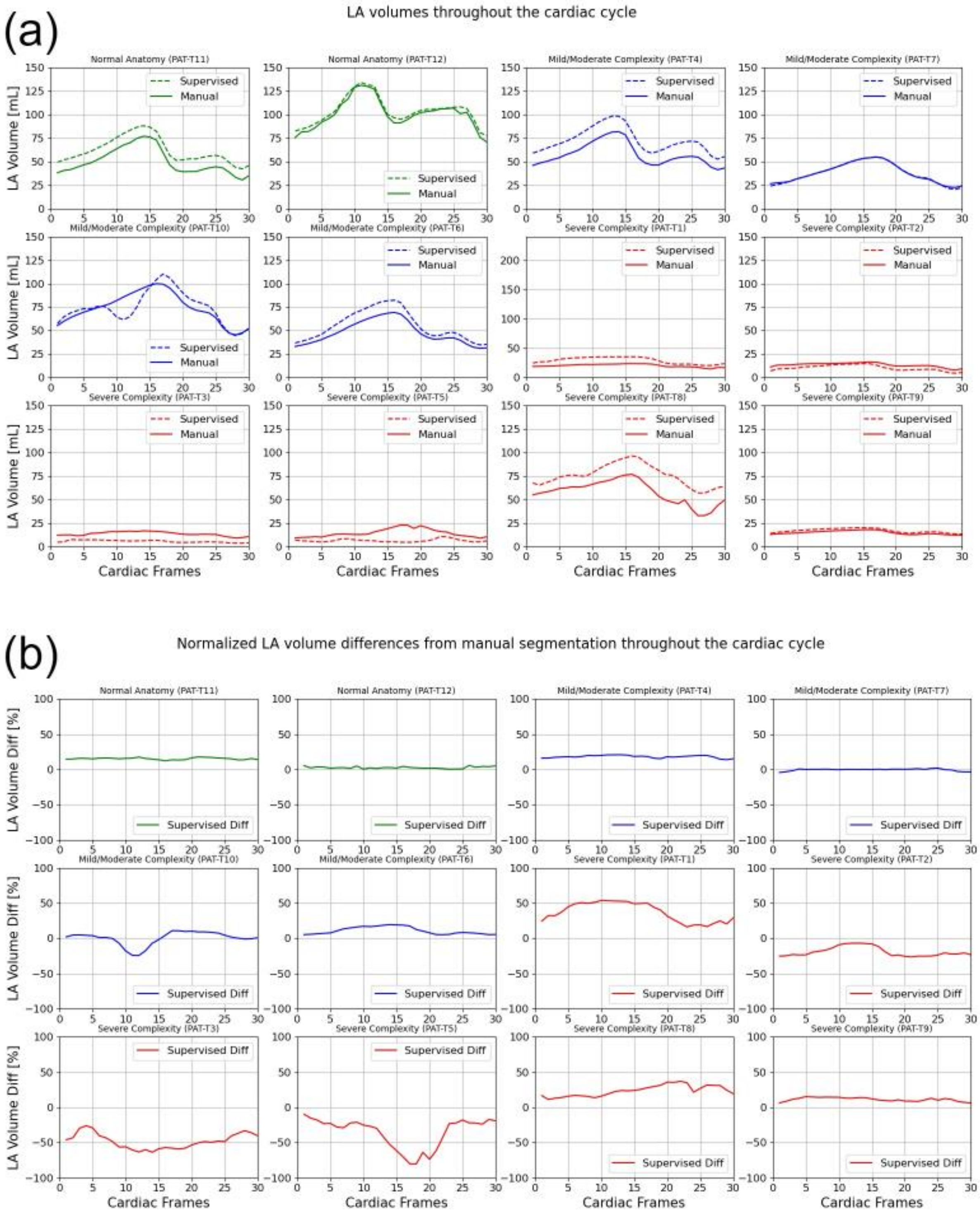

Supplementary Figure S4. Volumes of left atrium (LA) throughout the cardiac cycle calculated from 12 3D cine test datasets using the ground truth labels (manual segmentation) and the supervised segmentation method. (a) Values are given in mL, representing absolute volumes. (b) Values are given in percentages, representing volume differences between the manual and the supervised segmentation methods, normalized to the largest atrial volume per subject.

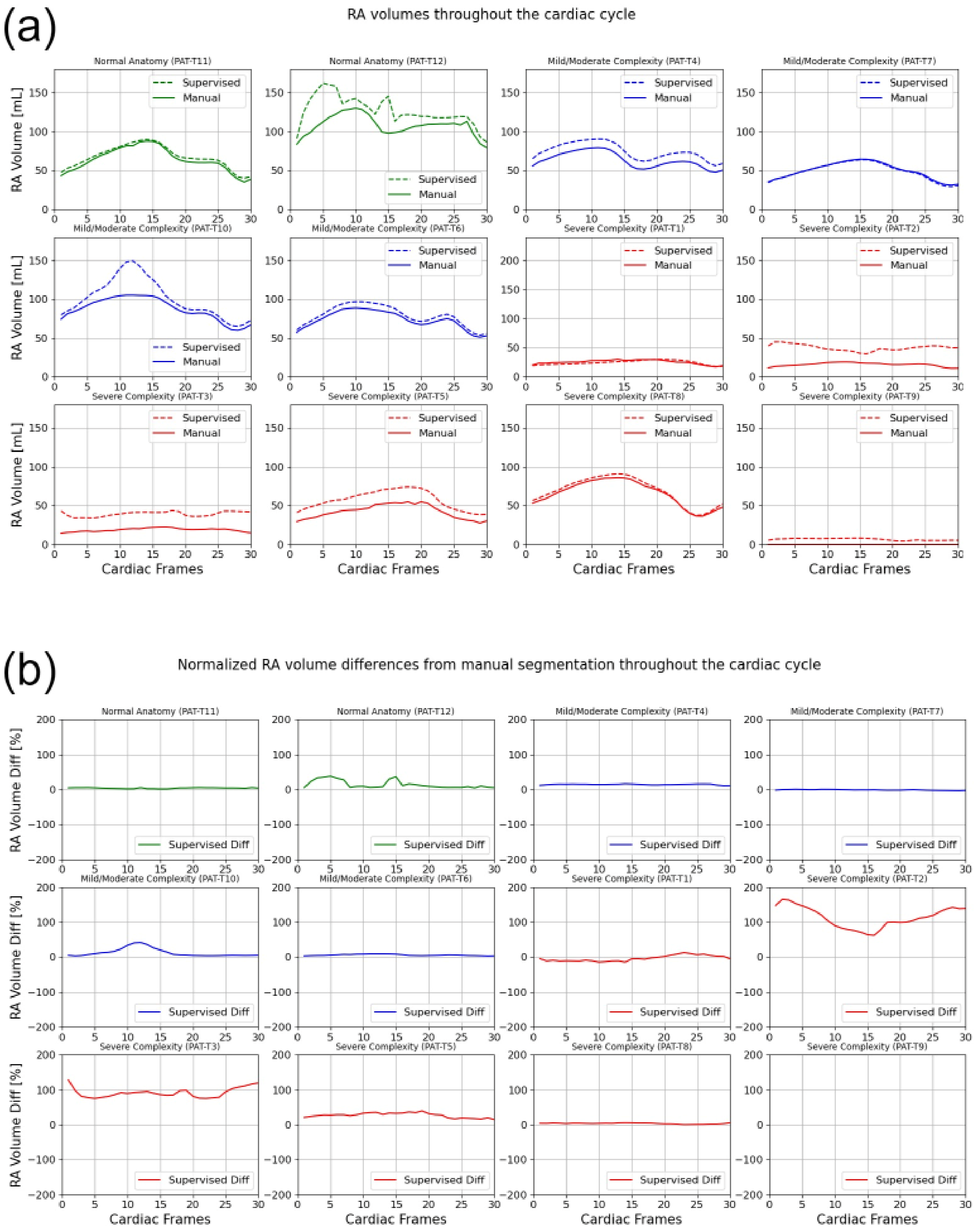

Supplementary Figure S5. Volumes of right atrium (RA) throughout the cardiac cycle calculated from 12 3D cine test datasets using the ground truth labels (manual segmentation) and the supervised segmentation method. (a) Values are given in mL, representing absolute volumes. (b) Values are given in percentages, representing volume differences between the manual and the supervised segmentation methods, normalized to the largest atrial volume per subject. PAT-T9 refers to a Fontan patient without RA.

2 SUPPLEMENTARY MOVIE FILES

2.1 Supplementary Movie File 1

Manual segmentation of 3D cine images acquired from a 16-year-old patient with atrial septal defect (subject PAT-T4 cf. Supplementary Table S2, mild/moderate category).

2.2 Supplementary Movie File 2

Automatic segmentation of 3D cine images acquired from a 16-year-old patient with atrial septal defect (subject PAT-T4 cf. Supplementary Table S2, mild/moderate category) using supervised DL-based segmentation method.

2.3 Supplementary Movie File 3

Automatic segmentation of 3D cine images acquired from a 16-year-old patient with atrial septal defect (subject PAT-T4 cf. Supplementary Table S2, mild/moderate category) using semi-supervised DL-based segmentation method.

2.4 Supplementary Movie File 4

Movie of a dynamic 3D model of the whole-heart and great vessels from a 16-year-old patient with atrial septal defect (subject PAT-T4 cf. Supplementary Table S2, mild/moderate category) generated from manual segmentation results.

2.5 Supplementary Movie File 5

Movie of a dynamic 3D model of the whole-heart and great vessels from 16-year-old patient with atrial septal defect (subject PAT-T4 cf. Supplementary Table S2, mild/moderate category) generated from supervised DL-based segmentation method.

2.6 Supplementary Movie File 6

Movie of a dynamic 3D model of the whole-heart and great vessels from a 16-year-old patient with atrial septal defect (subject PAT-T4 cf. Supplementary Table S2, mild/moderate category) generated from semi-supervised DL-based segmentation method.

2.7 Supplementary Movie File 7

Movie of a 3D cine sequence acquired from a 57-year-old subject with anomalous origin of the right coronary artery from the pulmonary artery (one of the subjects of the training set, mild/moderate category).
